# Public perception of carbon capture and storage: A state-of-the-art overview

**DOI:** 10.1016/j.heliyon.2019.e02845

**Published:** 2019-12-07

**Authors:** Pavel Tcvetkov, Alexey Cherepovitsyn, Sergey Fedoseev

**Affiliations:** a Department of Informatics and Computer Technologies, Saint Petersburg Mining University, Saint Petersburg, Vasilevskiy Island, 21st Line, 2, Russia; b Department of Organization and Management, Saint Petersburg Mining University, Saint Petersburg, Vasilevskiy Island, 21st Line, 2, Russia; c Luzin Institute for Economic Studies – Subdivision of the Federal Research Centre "Kola Science Centre of the Russian Academy of Sciences", Apatity, Fersman St., 24a, Russia

**Keywords:** Public perception, Carbon capture and storage, CCS, Review, Sustainable development, Natural resource economics, Willingness-to-Pay, Stakeholder analysis, Decision sciences, Well-being, Public opinion, Environmental change, Environmental science, Energy

## Abstract

Carbon capture and storage (CCS) is a technology enabling to use fossil fuels in a sustainable way. Therefore, it attracts much attention from the industrial sector, government authorities and scientific community. However, public awareness of the technology is extremely low, and the studies of the lay people's opinion have been launched only during the last decade. Taking into account the role of public support during the implementation of CCS projects, the authors would like to present herein their review of materials on this subject published during 2002–2018 (135 articles). As part of our review, we determined 9 key aspects forming the public perception of CCS. For each of the key aspects, we summarized the available results of the studies. Apart from that, we compared the CCS current status in different countries and provided a number of reasons for involving new countries into the fight against global warming. This work shows that most attention is devoted to CO2 storage; whereas its capture and transportation are poorly studied in terms of public perception. Wider development is required for the methodology enabling a transition from global rhetoric concerning global warming issues to the implementation of particular projects, namely, CCS. The issues related to public awareness of CCS are studied rather thoroughly, but no recommendations are provided regarding the establishment of an optimal database for the lay people. Numerous assessments of general public perception have been carried out. However little attention was paid to the regions with active projects, namely, to the factors considered the most important by the local public, and how actual project results meet their expectations. Therefore, despite an extensive scientific base developed over 17 years, further studies should be aimed at filling the existing gaps. This will enable to improve CCS attractiveness for the public, including the cases when it is compared with alternative low-carbon technologies.

## 1 Introduction

Global warming is a widely known problem discussed since 1960 after publishing of the Manua Loa Observatory's monitoring results, Hawaii [1]. During the past half of the century, several solutions were proposed, one of them is the implementation of the carbon capture and storage (CCS) technologies. The CCS technology involves carbon capturing at industrial facilities (gas and coal-fired power plants, cement plants, etc.), and its storage in geological reservoirs (depleted oil fields, saline formations, coal beds), or further use in production (Carbon Capture and Utilization - CCU).

During recent years, the CCS projects have shown that they can be economically viable, providing certain conditions are created and they can reduce CO2 emissions [2]. Although, just a while ago, their large-scale implementation was out of the question due to insufficient knowledge, specific risks, capital intensity, inadequate regulatory and legal framework, and absence of efficient mechanisms for carbon markets management.

Even though several countries (for example, China, USA, Australia) have managed to overcome such negative factors; today, implementation of the CCS projects slows down due to insufficient support from the government [3]. Therefore, it is important to involve new countries in the CCS technologies studies, and their capability for a large-scale implementation of such projects, to restrain annual growth of CO2 emissions. This especially relates to the leading producers of CO2 emissions, such as Russia, where the CCS projects are not considered, even in scientific papers.

Assuming the fact that the CCS projects are efficient, the experience of early countries that adopted the technology shows that negative public perception could be one of the barriers for its large-scale implementation, as experts or politicians usually have a neutral or positive opinion [4]. This is reasonable not only for CCS but also for all environmental technologies in general, as humans that are the source of pollution. Industrial operations themselves would not produce such a negative impact on the environment if the persons taking decisions strived to find a balance between economic efficiency and environmental safety, which is one of the fundamental principles of sustainable development.

Today, despite the fact that the CCS projects are implemented in different countries, the available scientific background is focused on two issues: studies of the CCS public perception, sometimes, in the regions where no pilot projects are implemented, but the public interest exists [5]; and global discussion related to the development of environmental technologies, namely CCS. At the same time, there is no connection between these two research groups, which would enable the transition from a global rhetoric to practice [6].

5–7 years ago, CCS was a young technology, and the scientists had to rely on the achievements in the field of public perception of more mature technologies (mainly, nuclear energy [7, 8]). Now the CCS technology has enough scientific background. Besides, until the present time, social studies, with some exceptions, were based on a predictive approach to the CCS public perception assessment. Today there is a long overdue need in the development of proactive social studies in this field focused on the justification of approaches providing an objective knowledge and creating a fair image of CCS technologies for public [9], including the countries, where a CCS project is only prepared for implementation. Based on the above mentioned, we consider it logical to step back and consolidate available knowledge in this field.

Therefore, the purpose of this study is to formulate the main principles of the CCS public perception development based on the global experience in the technologies perception assessment and extend it with Russian point of view on this problem for further implementation. Practically speaking, this will enable to develop a system of proactive public relations for balancing interests of all stakeholders, to achieve higher project efficiency and to minimize protest risk after the project startup, caused by misconceptions of locals [10].

The following part of this article includes 4 sections. Section 2 describes the selection of articles for the review, and distribution of scientific papers by various characteristics. Section 3 includes 10 subsections (Table 1) each of which is devoted to a separate group of factors that have the greatest impact on the public perception of CCS. The definition of these groups was carried out on the basis of preliminary analysis of the studies' results on the assessment of various factors impact on the public perception of CCS (see Appendix 1, column “Aim of the research”). Comparability and generalization of the results of these studies was possible because the key ideas underlying in most of them are interconnected. Section 4 has a similar structure to Section 3 and includes general outlook; and summary on each aspect of the CCS public perception. Section 5 highlights concluding remarks of the study and further research directions. They will be based on the results obtained herein.

**Table 1:** Structure of this article.

Subsection	Content and explanation
Awareness	In this subsection, we analyze the role of awareness in the CCS public perception. The subsection describes factors impacting on information sharing process, and possible ways of public awareness improvement. Public awareness of CCS implies the existence of fair knowledge about the nature of the technology, the causes, and consequences of its use, its strengths, and weaknesses, as well as benefits and risks.
Knowledge	Since CCS is not a thoroughly studied technology, we consider the problems of providing the necessary knowledge on its nature to the public. By knowledge, we mean the public ability to understand available information about global warming and climate mitigation technologies.
NIMBY	CCS is analyzed in terms of its susceptibility to the NIMBY (Not In My Backyard) effect, which may be defined as “social rejection of facilities, infrastructure, and services location, which are socially necessary but have a negative connotation” [11].
Benefits and risks perception	The key factors influencing on benefits and risks perception are described, and the relation between this perception and public attitude towards CCS is analyzed. By benefits/risks perception we mean the subjective judgment that people make about the characteristics and significance of consequences (positive or negative, respectively) for themselves and their environment.
Socio-demographic factors	The subsection determines the role of socio-demographic factors in CCS perception development. Taking into account the specifics of large-scale environmental projects, as well as the strong dependence of CCS project implementation on the mood of local public, this section considers the following aspects related to the social and demographic characteristics of the population: age, gender, education level, religion, expectations and values of people, as well as mentality and cultural specific.
Willingness to pay for CCS	Here we review the papers containing an assessment of public willingness to pay for energy rates growth due to the implementation of environmentally friendly technologies.
Trust	The subsection content can be described as follows: “trust is a psychological state comprising the intention to accept vulnerability based upon positive expectations of the intentions or behavior of another” [12].
Acceptance and Preferences between Technologies	Comparative analysis of public preferences related to the development of low-carbon technology packages, including CCS, or when several separate technologies are compared.
Governmental Policy and Interaction between Stakeholders	Analysis of the state policy influence on the CCS perception, and the role of individual stakeholders and their associations in public relations.
Cross-Country Outlook	Comparative analysis of the CCS status in different countries.

## 2 Materials and methods

### 2.1 Studies selection

According to [13], this study includes the following steps: search and selection of articles, data collection, literature review arrangement, reference list development, literature review writing.

Search for the literature on the CCS public perception were limited with the time interval of 2002–2018. This period covers all the history of such studies development. The articles were searched for in the databases Google Scholar (www.scholar.google.com) and Science Direct (www.sciencedirect.com). The inclusion of materials from Google Scholar (not included in the Science Direct) is explained by the fact that today many authoritative scientific institutions admit that high-quality research can be published in little-known journals, see [14]. Besides, the Science Direct indexes the most, but not all of the authoritative scientific journals. However, it should be noted that almost 90% of the selected materials were available in both databases.

For binary search, the following keywords were used: CCS, carbon capture and storage, CO2 geological storage, CCU, CO2 utilization; combined with public, perception, involvement, social, acceptance, communication, stakeholder, awareness. Selection of materials was based on the following mandatory requirements: language — English, scientific fields — social or economic studies, type — articles, review, reports. In total, 135 studies were selected. They are listed in the references among other materials added during the references development stage, and in Appendix 1.

It should be noted that even a detail search in the abovementioned databases does not enable us to conclude that this study includes a complete review of all scientific works corresponding to the above requirements. In addition, the information was collected from the selected articles, and structured (Sections 3.1, 3.2, 3.3, 3.4, 3.5, 3.6, 3.7, 3.8, 3.9 and 3.10) on the basis of the available experience in the field rather than stringent rules. Nevertheless, we believe that our review enables to analyze the most authoritative and important publications in this field, and achieve the goal of our study.

### 2.2 Studies distribution by sources, countries and years

This study reviews 135 articles related to the public perception of CCS technology. Nine factors (Table 1) that influence public perception of CCS were identified for the analysis. Table 2 shows how many of the reviewed articles consider these factors. The distribution of factors by the articles is shown in Appendix 2.

**Table 2:** The number of articles relating to the factors considered.

Factor	Number of studies	Factor	Number of studies
Knowledge	85	Socio-demographic factors	52
Acceptance of CCS and preference between technologies	83	Trust	42
Governmental Policy and Interaction between Stakeholders	81	Awareness	41
		NIMBY	38
Benefits and Risks Perception	79	Willingness to pay	12

The largest number of articles devoted to the influence of knowledge about the nature of CCS on the public perception, which is fair for almost all countries, because it is still little-known technology. The least number of references is accounted for NIMBY reaction and WTP (willingness to pay). On the one hand, this indicates the least degree of study of these issues. On the other hand, it is necessary to understand that these factors are part of the benefits and risks perception and they may simply not be highlighted in the reviewed articles.

In General, during 17 years, numerous materials were published in the field. This proves a high interest in this issue in the scientific community. Table 3 shows the distribution of the articles by countries and years. Active studies on the CCS public perception were launched during the last decade, and are implemented until now. One should also remember that 2018 is covered only partially herein. Cross-country research was presented as a separate group divided into countries as shown in Table 4.

**Table 3:** Distribution of the articles between countries in 2002–2018 years.

Country	2002–04	2005–06	2007–08	2009–10	2011–12	2013–14	2015–16	2017–18
Australia		1	2	3	1	1	1	
Canada				1	2	1		1
China				1		2	1	1
Finland						1		3
France				1		1		
Germany				1	3	2	1	5
Greece						1		
Italy				1				
Japan		1	1	1		1	1	1
Netherlands			4	6	5	1		
Norway								
Poland						1	1	
Romania								1
Spain				1	2	1		
Sweden		1				1		
Switzerland				2	5	2		
UK	1	1		2	1	7	1	6
US	1		1	5	2	3		
Vietnam							1	
Singapore								1
Cross-country		2	1	2	6	7	4	1

**Table 4:** Distribution of the articles between countries in the cross-country section of Table 3.

№	Country	Number of references
1	UK	12
2	Netherlands	9
3	Germany	8
4	Spain	6
5	Norway	5
6	Poland	6
7	Finland	4
8	Greece	4
9	Italy	4
10	Romania	4
11	Sweden	4
12	Belgium	3
13	France	3
14	Japan	3
15	US	3
16	Denmark	3
17	Czech Republic	2
18	Bulgaria	2
19	Australia	2
20	Canada	2

Table 5 shows the distribution of the articles by sources. Most of the articles are concentrated in several journals. Firstly, this is the Energy Procedia as it publishes the results of the largest international conference “Greenhouse Gas Technologies”. Secondly, this is the International Journal of Greenhouse Gas Technologies, which, as the name implies, specializes in greenhouse gas emissions. Other journals contain only a few articles on the subject, including 21 of 35 journals containing only one article.

**Table 5:** Distribution of articles between sources.

Source	2002–2004	2005–2006	2007–2008	2009–2010	2011–2012	2013–2014	2015–2016	2017–2018	Total
Energy Procedia			1	8	5	13	5	8	40
International Journal of Greenhouse Gas Control		1	3	8	8	4	3	2	29
Energy policy			1	2	2	4			9
Environmental Science & Technology	1	1		1	2				5
Climate Policy					2			1	3
Greenhouse Gases: Science and Technology					3				3
Applied energy						1		1	2
Energy Research & Social Science						1	1		2
Mitigation and Adaptation Strategies for Global Change		1			1				2
Risk Analysis				1		1			2
Risk Analysis: An International Journal				2					2
The Journal of Environmental Psychology			1	1					2
Renewable and Sustainable Energy Reviews						2			2
AGH Drilling Oil Gas							1		1
Australian Journal of Emerging Technologies and Society			1						1
Emory Law Journal			1						1
Energy							1		1
Energy & Environment	1								1
Energy & Environmental Science					1				1
Environmental Modeling & Assessment						1			1
Environmental Research Letters				1					1
Frontiers in Energy Research								1	1
GeoJournal							1		1
Human and Ecological Risk Assessment: An International Journal					1				1
International Journal of Global Environmental Issues			1						1
Journal of cleaner production						1			1
Journal of CO2 Utilization						1			1
Journal of Environmental Planning and Management							1		1
Journal of Experimental Psychology: Applied						1			1
Marine Policy						1			1
Sustainable production and consumption								1	1
Technology in Society						1			1
The Journal of Experimental Psychology: Applied				1					1
Proceedings of the Institution of Mechanical Engineers, Part A: Journal of Power and Energy				1					1
Other sources		3		1	2	1	1	3	11

**Figure 1:**
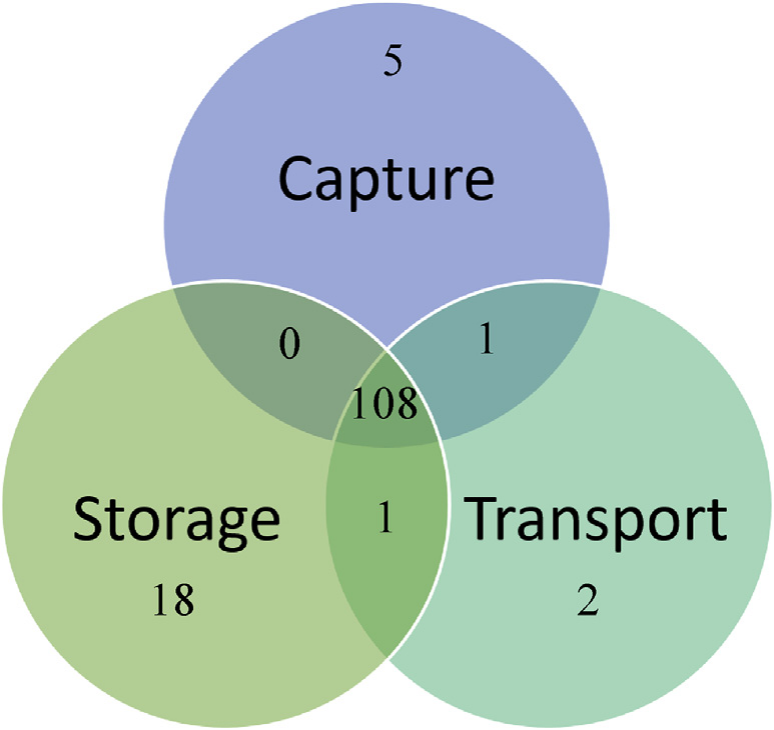
Distribution of the articles by technological stages.

Figure 1 shows the distribution of the studies between three key elements of CCS: capture, transport, and storage. Many social studies are focused on the technology in general, and even in such studies, much attention is paid to CO2 storage, which is a sole subject of 18 scientific works.

## 3 Results

### 3.1 Awareness

Almost all modern studies of the CCS public perception highlight poor public awareness of global warming issues and CCS [15]; although the level of general awareness has grown during the recent decades. Therefore, the first public reaction is usually negative [16]; however the same can be said about any little-known technology characterized by certain risks [17].

Recognizing this, the results show that in some regions the level of CCS public awareness is lower as compared with alternative green energy technologies. According to [18], in Australia 77% of respondents know about CCS, in the Netherlands — 84%, in Canada — 61%, in Scotland — 36%. It should be highlighted that a high public awareness in the Netherlands and Australia is explained by different reasons. In Australia, this is due to mass distribution of information on successful projects; whereas, in the Netherlands, this results from failure of the Barendrecht project, and the government's prohibition of the on-shore CO2 storages. This means that awareness does not show public acceptance of a project.

Despite such negative impression created by the information on closed projects, in most studies awareness is considered a basic condition for the CCS attractiveness improvement [19]. For example, characteristics such as “nature-like”, which is widely used now in scientific literature and mass media, can have a positive impact on public perception [20]. In general, this literature review enables us to conclude that the efficiency of information distribution depends on several factors, which are usually reasonable regardless of the specific characteristics of the target audience.

First of all, it is worth emphasizing that the efficiency of information distribution among the local public depends both on the level of trust in the project stakeholders [21], and the policy they are implementing (see Section 3.9); and on the quality of the presented information [22]. However, one should remember that the quantity of information is not equivalent to its quality. According to [23], the provision of additional information has a positive influence on the CCS public perception; but if we overload people with information, we create a distorted interpretation of the technology risks and advantages. Therefore, the distributed public information should be thoroughly selected, and the materials based only on emotional components should be avoided [19]. Scientifically based facts proved with the world's practice should be preferred [24].

Nevertheless, complete ignorance of emotional drivers of the CCS perception can be a mistake [25], as people are not always ready to assess rationally the advantages and disadvantages of a certain solution [26]. Sometimes it is necessary to show the best practices implemented by the leading countries [27]. According to [28], the most efficient sources of information on the CCUS projects are brochures describing project experience.

The brochures' effectiveness can be explained not only by the availability of information on active projects but also by the fact that they include a lot of graphics enabling people to understand complex concepts used in the CCS technology description [29]. For example, the paper [30] notes that storage is a special chain of CCS technology, which should be explained with illustrations, for instance, a picture of a sponge. This will help to avoid a wrong understanding of CO2 pumping into an underground reservoir, which is not similar to a balloon's inflation. However, we should remember that the ability to improve information perception with graphics is limited, and overloading of materials with illustrations can harm the perception of the text.

To minimize the risk of information overload, it should be reasonable to divide the information into separate parts and present them to the public one by one. However, if such activity is implemented in the form of training, then ensuring a required coverage can be a problem. A usually better understanding of the material is ensured with a small number of participants. Although there are successful examples of large groups training [31]. Nevertheless, for the purposes of public awareness raising at a national scale, such an approach will not be enough. This means that various information distribution methods should be used [32] with mass media support.

Mass media have a strong influence on the perception of any new tendencies and technologies, and their relationship with the existing problems, although the degree of such influence depends on many factors [33]. In [34] we see that the opinion of one of the respondent groups has become negative when they read an article describing the risks and uncertainties of the IPCC Special Report related to these technologies. On the other hand, the second respondent group read the same information in the form of an information booklet, and their opinion has not become worse. This means that mass media is an efficient method of public opinion control. However, we should pay much attention to the content of the provided materials, their relevance, level of trust in their source, and methods of the information delivery [35]. For example, such activities as press releases raise significantly the level of local public awareness and interest [36].

Another important issue is the assessment of public awareness of CCS, which is mainly based on questionnaires both for national and for local scale. Differences at these scales can be seen in the way the surveys are conducted. At the local level, it is possible to organize focus groups and workshops, where paper-and-pencil and face-to-face interviews could be conducted. At the national scale, as a rule, online tests and telephone surveys are used, which are carried out by specialized agencies. Obtained results are a valuable source of information that allows predicting future public opinion about CCS, for example, with using Theory of Planned Behavior.

### 3.2 Knowledge

The CCS understanding depends much on a public understanding of the global warming issues, CO2 emissions growth, and potential alternatives of the emissions reduction, as well as the potential of such activities for the economy [37]. Misunderstanding of the CO2 emissions concept creates the wrong idea about CCS technologies [38].

In addition, the level of background knowledge and awareness of the CCS technology, before its public discussion involving more detailed information and various points of view, does not always show a probability of its approval or disapproval [39]. Nevertheless, when a person decides on the CCS approval/disapproval, a general level of knowledge and awareness plays an important role [40].

At the same time, an organization of public discussions should include distribution of information concerning the technologies fundamentals, which can be described later, among as many members of the local public as possible [41]. This will enable to minimize the negative impact of the contradictory information from the Internet. We should also bear in mind the public's limited attention [30] resulting from a tremendous amount of information daily received. Therefore, the experts should make additional efforts to raise public interest in familiarization with the material. Without it, opinion polls will not produce the required results, as polling of the respondents who do not have minimum knowledge of the subject is not a reliable source of data [42].

If the target audience does not have the necessary knowledge about CCS, it is important to provide the information, which enables to look at such projects from different angles, including brief reviews of low-carbon technology alternatives, their relation to the global warming issues [22].

For instance, despite a detailed description of CCS, the information given in [43] was not exhaustive for people unaware of the low-carbon technologies, as this information showed only the opinion of NGOs, which disapproved the CCS implementation in Quebec. Several other materials show the same situation.

On the other hand, today, there are no CCS studies that enable to definitely determine a complete list of information materials required for the description of the whole situation and alternatives. Nevertheless, it is important to provide at least the data from various stakeholders, and on several alternative technologies to the respondents. This will enable to get reliable perception results. However, information overload can lead to the lay people's misunderstanding of the key issues [23, 44]. We can improve the understandability of the CCS materials by making comparisons with clear and obvious things, or natural events [34].

The difficulty of objective CCS knowledge presentation can be explained by the fact that the technology has not been studied enough, for example, the issue of CO2 underground behavior at extremely high pressures and long-term migration. This can be a reason for misunderstanding. One of the most commonly held misconceptions is an idea that CO2 pumping into the underground reservoir is similar to balloon inflation [30].

According to [45], education in the CCS and global warming issues can be effective only if it is implemented before a person develops his own opinion on these issues. If a person has a certain idea of the CCS issues, in some cases, it will be rather difficult to change it [34, 46]; but this is possible providing the necessary communication channels and approaches to the target audience are found [39, 47]. Nevertheless, it is believed that a change in the initial opinion about the CCS from negative to neutral, or positive, which occurs after such training, can be driven by a focus on certain CCS advantages, rather than a general description of the situation [42].

Development of knowledge about CCS, global warming issues and alternative low-carbon technologies is a multifaceted objective, which can be complicated by false hopes and beliefs based on superficial knowledge on the subject. That is why stakeholders should use a proactive approach towards the provision of information on CCS development issues.

### 3.3 Not in/under my back yard

The NIMBY effect is produced by various factors, including the known risks, values and a sense of unfairness. The effect is a new concept for the CCS technologies, and, in general, it is a normal response to a potential hazard located near a permanent residential area.

The paper [48] shows that in Japan people were rather tolerant or neutral towards the CCS ideas and its role in the governmental programs on CO2 emission reduction until the moment when it comes to implementation of particular projects (before the accident at the Fukushima NPP). This is how the CCS comes in practice, and the risks and the public concerns become real.

The same is proved in [49], but in relation to the cities of Alkmaar and Bergen. The research compared the public attitude to the CCS in general, and to the implementation of certain projects near their cities in particular. The results showed that most respondents understand much better the risks of local projects.

According to [9], despite a high CCS approval rating, the same situation exists in China, where 48.4% of respondents would prefer if CO2 storage was located more than 100 km from their home, 23.9% of respondents approve the location of CO2 storage within a radius of 100 km, and only 3.5% approve a radius of 10 km.

Even in Australia, the world leader in the CCS technology development, the studies [50, 51] showed that 42% of 1273 respondents would be concerned if CO2 storage was located near their city, and the respondents (41%) also added that the CCS is a temporary solution for greenhouse gas emissions. Only 21% of respondents were confident in the technologies safety and strict control of the projects.

The analysis enables to conclude that there are no standard solutions guaranteeing the elimination of the NIMBY effect. Raising of public awareness, improvement of the technology image, provision of additional high economic incentives, and other similar methods can produce an opposite reaction and a suspicion that the stakeholders hide some important information on the real project risks.

Nevertheless, not everybody has the NIMBY reaction, and under certain conditions, it is possible to find a compromise. According to [52], the NIMBY reaction can be mitigated by changing of separate CCS process stages. For example, respondents prefer the construction of a biogas-fired plant in their cities rather than a gas-fired plant. However, the reluctance to have CO2 storage and a pipeline system nearby remains. The results are similar to [53], where, however, different results were obtained for a gas pipeline system, which did not bring a protest.

According to [54], despite a rather high percentage of the NIMBY reaction in Indiana, the respondents’ opinion was mitigated by a detail description of economic benefits from the project implementation near their residence, for example, job growth, fiscal loosening, and other economic incentives. At the same time, the information on such risks as CO2 leakage, induced seismicity, explosions, and groundwater contamination increased the NIMBY reaction dramatically. In addition, the reaction is increased when the local public does not approve their local authorities, and their project implementation policy. Eventually, this can lead to the loss of trust in other project stakeholders [55] and refusal to support the local project.

Whereas in Russia, speaking about a large-scale implementation of CCS-EOR, the NIMBY reaction is not such as acute issue due to the following factors. Firstly, there are quite many gas pipelines here now, and some of them are laid through the cities. Additionally, a heat supply system consists of pipelines. In other words, if people do not protest against existing similar infrastructure, then, probably, the risk of protest against CO2 pipeline will be minimal. Secondly, according to global practice, the most negative NIMBY reaction appears in the case of CO2 storage near residential areas. The most prospective oil fields for the CCS projects implementation are located in Siberia, far away from both large cities, and small settlements.

### 3.4 Benefits and risks perception

Benefits and risks are two sides of the same coin from the economic point of view. However, when we talk about social phenomena, such as public perception of CCS, factors impacting and depending on benefits and risks perception are different, although, for example, trust in stakeholders can impact on both of them [56, 57]. Besides, the strength of their impact on CCS perception is also different. According to [58, 59] and many other studies, benefits perception have a stronger impact on CCS acceptance, than risks perception, regardless of a serious concern about the immaturity of a storage technology [60].

On the one hand, this means that it is necessary to focus on a description of positive aspects of the technology as they have the greatest impact on the public. On the other hand, it requires a detailed study of the information base related to the risks of CCS technologies, which are still not known completely. This uncertainty, along with insufficient knowledge about the physical-chemical properties of carbon dioxide [61], strengthens a negative image of the CCS, which raises public concerns [62] and increases the likelihood of a protest potential [63]. Similar ideas are described in [34], where it is stated that despite the prevailing impact of the project efficiency on general acceptance, such factors as the risk of CO2 leakage from the storage facility change the attitude to CCS technology. Similar conclusions were drawn in [64], which states that it is the process risks that are of primary importance for the public.

Besides, there are examples where local incidents have a negative impact on the public perception of CCS. For example, the explosion of a gas pipeline in Belgium in 2004 increased public unrest in relation to the reliability of the CCS process chain, namely a transportation component [65]. A similar situation is observed in Japan, where until 2011 people were quite loyal to the offshore CCS and more concerned about on-shore projects. However, after the accident at the Fukushima NPP, people began to look more negatively at prospects of the on-shore and off-shore CO2 storage due to possible leakages caused by earthquakes [66], although the overall CCS perception has changed insignificantly. In fact, the CCS risks are not significantly higher than those of other new technologies [4]; however, due to insufficient development of the technology itself and the novelty of the geological CO2 storage technologies, which have appeared in some countries only recently, such risk generate there the same concerns that were observed in the pioneering countries at the beginning of the century [67].

Contrary to the significant role of technical factors, the study [57] shows a significant impact of socio-cultural factors on the benefits and risks perception. And, in spite of the fact that the number of such factors can be very high, and they can vary depending on the region assessed, an attempt was made in [68] to identify the key ones:

- “risks perception: uncertainty avoidance, determined as “the extent to which members of society feel uncomfortable with uncertain, unknown, ambiguous, or unstructured situations” and society's short-term or long-term orientation;
- benefit perception: uncertainty avoidance, long-term orientation, and inequality of power distribution in society (power distance)”.

The study [30] implemented in Switzerland also notes that, under certain conditions, concern about socio-economic factors has a stronger impact on the risks perception than technical factors. Concerning the analysis of socio-economic factors, we would like to highlight the work [69], where the first attempt was made to reconcile the economic parameters of the CCS implementation and public perception. The authors managed to achieve two important results. Firstly, they showed that the CCS projects implementation could influence on the public (country) welfare. Secondly, they showed that a possibility to use CO2 could positively influence the national industry. In other words, only detail analysis of the technology risks and benefits will enable to take a balanced decision [70].

### 3.5 Socio-demographic factors

Social sciences helped considerably to widen our understanding of the CCS projects risks. This is associated with different points of view on such risks existing among scientists, technical experts and community, especially people that live in the regions where projects are planned [71], including the impact of social, cultural and demographic factors [72, 73].

The socio-demographic group of factors is extremely large and, depending on the features of a particular group of people, the impact of individual factors on their perception of an idea can change. Despite this, this section describes some examples of the key socio-demographic factors influence on the perception of the CCS technologies.

According to [47], in terms of the CCS public perception, an important role is played by personal perception, expectations, and values of people, rather than care of the national economy. In other words, during interaction with the public, we should, first of all, take into account their mentality [74, 75] and cultural specific [73], and draw parallels between their personal needs and CCS global impact on climate change, see [76]. This enables us to draw a conclusion confirmed in [77] that interaction with the public should be carried out after a detailed study of their internal organization, specific motivating factors, expectations, and goals.

It should be noted that the age, contrary to expectations, is not a factor determining the CCS perception [50, 73]; although, in some earlier studies, the perception of innovative energy technologies was associated with the age of respondents [78]. This can be explained by a popular opinion that a human conservatism increases with age. In general, it is obvious that people of different age groups have different thinking, and different approaches are required to influence them [79].

We should also bear in mind that men are more likely to perceive new technologies and participate in their public discussion than women, which is confirmed by numerous studies [50]. Additionally, men have a more tolerant perception of the risks where the economic potential exists, while women are more concerned about safety [79]. The same is true for CCS technology, as shown in the national survey of UK residents [80] and some other studies. Similar ideas are outlined in [81], which shows that masculine societies are much more focused on economic growth than feminine nations, which increases negative impact on the environment. These results enable us to state that cultural features play important role in the CCS perception, even when it comes to expert assessments [50]; however, one cannot draw conclusions basing only on such factors.

Religion has also a certain influence on the CCS perception. For example, according to [82], atheists have the most favorable attitude to the CCS as they do not believe in the afterlife. Christians are somewhat less loyal to CCS technologies; however, they are ready to support them if they have a positive effect on human well-being. Muslims demonstrate the most problematic CCS perception due to the peculiarities of their religious beliefs. In general, both Christians and Muslims have a low perception of the global warming issues and greenhouse gas emissions driven by their belief in the afterlife and divine intervention. The extremely limited number of publications in this field complicates the development of approaches to various religious groups, which is especially important for Russia, where there are representatives of many religions, and the predominant religions are Orthodoxy and Islam.

Religion determines a person's position in society and also determines its place in global trends. However, individual responsibility for reducing CO2 emissions is a little-discussed issue [83]. Although a lot of people understand the importance of energy conservation, recycling, and other environmental activities, there are clear problems with the establishment of a relationship between global warming and the daily life of a person, according to [84, 85].

Quite the opposite results are shown in the paper [86], where the discussion about the perception of geological CO2 storage, on the contrary, led to people's descriptions of their personal impact on the environment, and of the fact that the need for CO2 storage is associated with not enough sustainable ways of our life. On the one hand, such differences can be explained by the difference in the methods of discussions. On the other hand, the results, probably, were influenced by certain national factors, since [86] is a cross-country survey, however, there is no evidence of such dependence.

Perception of global warming issues, understanding of a human role in this process and development of an objective view of the low-carbon technologies prospects, including CCS, depend on the education of respondents [50, 64]. Therefore, probably, we should consider the implementation of an educational strategy for sustainable development that begins in school, and could be a part of a national "green" policy [87].

### 3.6 Willingness to pay for CCS

Theoretically speaking, users of electricity may not care about what resources it is generated from, as it is a product with stable quantitative and qualitative characteristics, which often remain permanent. Additionally, in some regions, large power plants are located far from any cities, which enables to minimize their effect on the environment. However, there is a number of evidence of altruistic behavior of the lay people, so-called "Willingness to pay for CCS", i.e. voluntary consent of the public to electricity and/or heat rates increase for implementation of the CCS projects, in case of capturing CO2 at power generation facilities.

An objective fact is that at the stage of demonstration projects, the CCS should be implemented with substantial financial support from the state without charging people. However, large-scale distribution of the CCS technologies is closely associated with the public willingness to increase electricity rates, which can adversely affect the perception of the technology [88].

According to [89], in Germany, respondents were ready to pay 15.9% more for electricity in case of a 10% increase in the CCS power, and 26.3% more in case of a 10% increase in green energy; although these results differ from the studies conducted earlier [90], which showed that 25% of respondents are willing to pay 1–5% more for energy produced from environmentally friendly sources, 16% of respondents are willing to pay 6–10% more. A significant proportion of the respondents either are not ready to pay more (33%) or could not give an exact answer (15%).

The [91] shows that, based on altruistic considerations, the Japanese are ready to pay a certain price for electricity from certain sources. For 1% growth of the renewables share in their energy consumption, they are willing to pay 11 yen, thermal power with CCS — 4 yen, and 1% reduction of nuclear energy share — 14 yen.

According to the research [24] conducted in the UK, 90.3% of respondents after the workshop on low-carbon energy technologies (88.6% before the workshop) are willing to pay maximum 50 pounds per quarter (2.2% of their monthly salary). In general, another research conducted in the UK [80] shows that people tend to be more negative about technologies that increase the electricity rates, which, along with certain CCS risks, is a significant barrier.

As for Russia, it is safe to say that willingness to pay will be much lower, because the average salary in the country is 39,000 rubles, which is equivalent to 446 pounds as of August 23, 2018. In this regard, it is important to determine whether lay people are willing to allocate enough funds for large-scale implementation of projects, which largely depends on different technical, political and economic factors [92].

Such results can also be useful in case of freedom in choosing an electricity supplier and can be an effective tool for an energy company to gain consumers’ trust. In Russia, such a situation is possible in the central and eastern parts of the country, where there is no developed network of gas pipelines and there are various options for energy supply.

Willingness to pay can strongly correlate with willingness to accept [93], which determines the importance of this aspect of public perception for the purposes of this study. If we have information about a potential project cost and the level of average salary in the region of the project implementation, we can preliminarily assess the prospects of the project approval by the local public.

### 3.7 Trust

Trust is an effective tool for the popularization of CCS, but only if the public understands the goals of the industry, NGOs and government authorities [49, 94] under the project, and these goals are not conflicting [63]. Otherwise, people will rely on their own judgments, philosophy [95], knowledge and experience [96], ignoring information from other sources.

The research [97], based on the analysis of the opinions of 811 respondents from Barendrecht, shows that local residents had a negative attitude towards the current CCS project, as the town council and the respected local activist group "CO2isNee" could influence on the project implementation only to a small extent. Whereas the level of trust demonstrated by the local public in relation to the main project stakeholders (national government and Shell) was much lower. Partially, this resulted from the fact that they did not have enough experience of successful joint interaction, the importance of which was highlighted in [98].

In view of this, we can conclude that there is a need for a proactive policy in the field of stakeholders’ interaction with the local public, which should be launched before project implementation begins [99]. At the same time, in the context of globalization and information availability, this policy should be transparent and reliable, as any negative consequences of the project implementation in any country will immediately become available to the public [100].

The experience obtained in Barendrecht also shows that the choice of trusted organizations also important. Such organizations can mediate between the local public and unknown project stakeholders. This will increase the level of the people's trust in the information received, and establish feedback for timely response to misunderstanding [38]. Similar results were obtained in the study [50] conducted in Australia. It showed that in all groups of respondents the Commonwealth Scientific and Industrial Research Organization (CSIRO) had the highest level of trust in comparison with other organizations (68.6%). The Australians also tend to trust scientists who are not working for the government, and the National Government by itself had only 20.4% of trust.

Denial of the fact that people demonstrate a higher level of trust in some organizations, and lower — in the others can lead to a denial of the proposed idea and technology importance [97]. In addition, in countries with a developed system of environmental NGOs, people can trust them more than the government and companies. According to [101], people consider NGOs the nature protectors and people's servants, unlike companies, whose motives are not always clear to them [102]. Therefore, development of a public relations strategy needs the involvement of as many experts from the non-government sector as possible [64].

The related ideas are also described in [101], which shows that the level of public trust in the environmental arguments of the industry representatives is significantly lower in comparison with NGOs. However, the opposite is also true: the industry's arguments about the economic aspects of the project implementation are perceived better by the community than the NGOs' ones [103, 104]. Logically speaking, this perception can be explained by the fact that it is economically unsound for the industry to honestly and openly provide information, which poses a potential obstacle to its activities. Whereas the NGOs' purpose is to maximally reduce a human impact on the environment regardless of economic effects.

At the same time, the public trust implies its involvement in the implementation of the CCS project at the earliest stages [105], and interaction with other stakeholders should include, but not limited to, risk communications, as confirmed by a number of projects in Canada and the USA [106]. This can have a positive effect on the public trust in the project stakeholders, although it does not guarantee support of the CCS technologies [107]. We should also note that competence trust in the CCS-related issues is an important factor [101, 108], which requires the provision of certain proofs from stakeholders, and selection of such experts, whose opinion the public will consider [65]. Trust in experts in case of CCS technologies is much more important than in case of alternative low-carbon technologies, as the CCS effect is not apparent immediately; therefore, most lay people have to rely on the specialists’ opinion on a positive long-term effect [64].

As for the CCS implementation in countries where public awareness of such projects is minimal, it should be noted that, unlike the first CCS projects, today we have already experience of such projects implementation in the world practice. This can increase the attractiveness of the technology; however, it requires trust in the project stakeholders, who can adequately describe their experience, and prove that they can ensure fulfillment of their promises under the project [95].

### 3.8 Acceptance and preferences between technologies

The studies of the public perception of CCS are aimed to identify the factors that influence its acceptance or rejection. According to [109], acceptance by itself is a ‘behavior that enables, supports or promotes an energy technology, in contrast, to open and expressed resistance to it, while acceptability is referred to as an attitude or evaluative judgment towards an energy technology’. However, the paper [110] shows that the terms “acceptance to” and “support for” are not equivalents in terms of environmental technologies. The former means a passive form of a technology approval (for example, approval of the corresponding research financing [111]), while the latter implies active support of project implementation. This enables us to conclude that positive results of acceptance assessment mean not so much public support as its willingness not to protest against a project. Such a situation can be rather shaky, and result in mass protests, especially, if people are aware of the technology risks, and do not have enough information about its benefits [63].

Development of a protest potential can originate from both the essence of the technology itself and the absence of alternative options that can be offered to people. In this regard, the studies aimed to compare the degree of support for various low-carbon technologies become important. For example, the paper [112] shows that having known advantages and disadvantages of the CCS, the respondents are inclined to compile a portfolio of several technologies, including the CCS, rather than choose one particular low-carbon technology. At the same time, in such energy portfolios, the CCS can take both a small [113] and a significant share [112].

Many papers [9, 114, 115] note that renewable energy is better perceived by the public than the CCS, even after a detailed study of the technologies. However, most of these studies were carried out at the initial stage of the CCS establishment, when the world practice did not have enough project experience. For example, a survey of citizens in the USA, UK, Sweden, and Japan [116] showed that among a significant number of alternative low-carbon energy technologies, the CCS has one of the lowest priorities along with the nuclear power, although the number of overt opponents of the CCS is slightly lower. Today these results are not unambiguous and are refuted by a number of local and national studies.

Nevertheless, there are a number of factors that are still relevant for comparison of the CCS and renewable energy and cause public concern. According to [88], a high proportion of respondents from different parts of the EU fear that the CCS development can lead to budget cuts and delay in the renewable energy development, which is more preferable [117]. It should be noted that the world scientific literature does not have any reliable evidence confirming such substitution, as these technologies are not interchangeable, although they are both aimed at the energy sector greening. However, it is possible to redistribute funds in case of refusal from the CCS technologies. In a number of studies, we can see also public concerns about the fact that the CCS will not enable to solve the problems of climate change and energy [16], which can be neither proven or completely refuted at the current stage of their development.

Another study [118] conducted in China shows that alternative energy is slightly more preferable than the CCS. At the same time, the respondents believe that CCS perception can be improved in comparison with the perception of renewable energy, if the government, industry, and NGOs take the following actions: strengthen international cooperation, organize public events and training workshops, develop regulatory framework for the industry control, and develop a system of incentives for various stakeholder groups.

Another area of studies is the assessment of the CCS perception with account to changes in conditions of individual process stages implementation [113]. For example, the paper [53] shows that coal-fired power plants are less preferable than biomass power plants, or capture at industrial plants. Another paper [52] shows that using a biogas-fired plant as a capture facility is more preferable than using a gas-fired plant. In general, public preferences related to capturing facilities are explained by not so much the CCS technology issues as the problems of the existing energy infrastructure. Nevertheless, this factor also impacts on general and local acceptance of the CCS projects.

The paper [53] shows that the type of CO2 storage selected under the CCS project is also important for people. Thus, CO2 storage in depleted oil fields is more preferable in comparison with storage in saline formations. This fact enables us to consider the option of providing economic incentives to local public funded with the profit from the implementation of the project. This approach can slightly increase a payback period of the projects, but, at the same time, it will increase the likelihood of support from local residents.

There are also examples of a reaction when the CCS idea itself is supported by the respondents; however, when it comes to particular options of its implementation, for example, as part of gas and coal power plants, public preferences can change to negation [119]. We should note that the opposite situation can also happen. The paper [120] describes a significant work done to improve the public perception of CCS under the Otway Project. According to the authors, one of the reasons for public approval is its favorable attitude to the development of the gas infrastructure.

Additionally, in recent years, the studies related to CCS and CCU comparison have begun to develop. According to [26], people tend to prefer beneficial use of CO2, rather than its storage in sub-seabed or saline formations. However, in the context of socio-economic studies, the issues of effective CO2 usage should be considered with due caution. Firstly, CO2-EOR, despite the "storage" stage, implies an economic effect obtained by increased mining rate, which is not always communicated to the respondents. Secondly, practical implementation of many CO2 usage alternatives, such as methanol or fuel production, is impossible under current conditions due to the immaturity of the technologies and extremely competitive market.

For instance, despite a small number of respondents and the university-based nature, the paper [121] determines some results obtained for the public perception of possible alternatives to geological CO2 storage. Thus, the most preferred way of CO2 usage is methanol production, whereas the most efficient process chain of CCS-EOR is perceived as one of the worst alternatives, second only to the CCS without the beneficial use of CO2.

Despite a number of methodological issues, such studies are up-to-date and required for determination of public preferences. They are also useful for creating a general image required for some people to understand the CCS technologies [122]; otherwise, they will search for information about alternative uses of CO2 in unreliable, and often inconsistent Internet sources [62].

During the review of scientific papers related to the comparison of CCS with alternative low-carbon technologies, we would like to note that in early studies the technologies are widely compared with the nuclear power because they have similar risk levels. Today scientific papers do not use this single comparison. Firstly, this is explained by the fact that the CO2 storage phase alone arouses serious concerns due to insufficient knowledge, although the CCS has not yet led to cataclysms, unlike nuclear power plants [123]; therefore discussions about their risks are much more of theoretical nature. Capture, transportation and beneficial use of CO2 are generally perceived by the public either neutrally or positively.

Secondly, over the past 10 years, much progress has been made in the studies related to technology safety, and a lot of experts, whose opinion the lay people consider, have appeared. Despite the fact that up to now the information on the CCS projects safety is not exhaustive, this enabled to develop a scientific framework, which, to a certain extent, overlaps other environmental technologies, but remains independent. Although there are some exceptions, for example, the paper [80] compares the perception of wind energy, nuclear energy, and CCS. And, while a decided preference for wind energy over the other alternatives was quite predictable, the choice of nuclear energy as a safer technology than the CCS was unexpected.

### 3.9 Governmental Policy and Interaction between stakeholders

Being an innovative technology, the CCS can be developed only with support from the state, which will enable these technologies to establish at the market [124, 125]. Comparison of the CCS projects public perception in 4 USA states [126] showed that a confident governmental policy of greenhouse gas emissions reduction and provision of information on the problem importance to the public enabled to receive a more favorable attitude from the citizens during the survey, in spite of the fact that they expressed their concerns about economic, technical and political risks. The paper [127] also notes that the state policy in the field of CCS development should be developed for a long term, as its presentation as an interim measure can negatively affect its perception, both by the public and other stakeholders. In the absence of such policy, even the interest in the implementation of the projects expressed by numerous stakeholders cannot produce the expected positive result.

According to [104], there is little difference in perception of the global warming issues by the CCS stakeholders from different industries and organizations. The survey included 142 respondents (North America, Europe, Japan) from the power industry, oil and gas companies, NGOs, government and educational organizations, as well as a number of other carbon-intensive industries. The results showed that global warming issues and CO2 emissions growth are urgent global problems, which is difficult to solve with the available technologies. At the same time, most respondents believe that such environmental technologies as CCS will find ways for a large-scale expansion during the next 10–20 years due to the presence of important drivers of their development. The same situation was observed in the field of renewable energy at the end of the last century when the cost of renewables was much higher than the cost of traditional energy. However, active state support in a number of countries enabled to reduce the renewable energy generation cost multifold, and achieve a large-scale implementation [128].

Consequently, one of the key challenges to improving public perception of the CCS is the consolidation of the government, industry and NGOs' efforts [129]. However, an organization of such interaction based on the principles of transparency and openness [130] is a much more labor-intensive process than the interaction of one of the stakeholders with the local public, due to different points of view on separate elements of the project [126]. The public perception directly depends on the effectiveness of interaction with stakeholders, and how they share responsibility [131], and also whether they have similar expectations about the potential effect of the project implementation [108]. For example, the paper [132] notes a clear skepticism of the local community about the company building a CO2 pipeline, as its impact on the residents’ life has not been clarified. Nevertheless, this disapproval could be mitigated by providing the local people with comprehensive information on the fulfilled safety measures, and a detailed description of the impact that has the gas pipeline construction on living conditions.

In addition, according to [101], the public tends to trust political decisions if they are sure that all stakeholders had an opportunity to express their opinion on the project, and their interests were not restricted. Public discussions of the CCS technologies enable to show a socio-economic and technical nature of such projects [133], as well as the fact that these technologies are one of the possible steps to reframing the society's energy policy and a long-term transition to clean energy [84].

According to [134], the public discussions based on the equality of votes and freedom of expression have a positive influence on the perception of a particular technology and enable to formulate a package of measures that will increase its attractiveness, as well as assess uncertainty and some risks related to the technology implementation [62]. At the same time, if government authorities, as well as the neighboring countries, implement an active policy in this area, the issue also takes a political context [135], which can strengthen public trust due to the importance of collaborative decisions.

In Russia, the government is actively pursuing a policy of import substitution due to the imposed sanctions. In particular, it covers also renewable energy, where a regulatory and legal framework was developed to enable renewables to occupy a dominant position on the energy market. However, due to insufficient interaction between individual stakeholders, Russia lacks production capacities to achieve these goals, and the public is not involved in the implementation of clean energy projects. Such factors challenge the effectiveness of clean energy long-term development [136].

### 3.10 Cross-country outlook

The difference in the national context of CCS perception is formed under the influence of numerous factors, ranging from geographical location and ending with the experience of the public interaction with the state and large energy companies. Accordingly, the main objective of this section is to review similarities and differences in the trends of the CCS public perception.

The CCS perception can have a pronounced national context. For example, in some countries of Western Europe [26], and in the USA [137], people are quite loyal to the seabed CO2 storage. The same situation is observed in the Nordic Region, where the CCS on-shore projects failed under the public influence [135]. On the other hand, in North America, the situation is the opposite, and people prefer on-shore storage facilities.

Another example of different CCS perception due to national differences is the NGO's of Norway and Germany. The former actively support CCS as an efficient method of the fight against global warming. The latter criticize these technologies because of potential risks and low efficiency of common storage methods [72].

The factors determining risk and benefit perception also differ. According to the study [138] conducted in Switzerland, the key factors are socio-economic factors related to the unsustainable nature of individual stages of the CCS technology. Probably, this is determined by the national internal policy and energy strategy, which is aimed at the reduction of the nuclear power share. As part of this process, the transition to gas-fired power plants can be an intermediate stage of sustainable development, which involves also CCS.

In contrast to [138], according to the study [57] conducted in Canada, level of trust in the state authorities, companies and NGOs has maximum impact on the risk and benefit perception. Despite various impacting factors determined by the national context of technology development, the technology perception is favorable in Canada, providing certain government support [139], and in Switzerland.

In Canada, this can be explained by the fact that one of the drivers of the national economic development is fossil fuels, especially in such provinces as Alberta and Saskatchewan. In other words, people have already got used to the presence of mining companies and transport systems, which is a positive factor for the public support of CCS development. This fact is confirmed in [140], which shows that the CCS is perceived more loyally in those regions where mining companies have been actively operating already, in particular, oil and gas companies. On the one hand, this is a way to improve the environmental situation in the region, and, on the other hand, it will increase the economic efficiency of the industry in the region.

China produces the largest portion of the global CO2 emissions and has a number of CCS projects on its territory. At the same time, the paper [64] notes that a low level of awareness and limited attention to environmental issues characterize the lay people of China. These features adversely affect the CCS perception, although there is no apparent confrontation against this technology. The paper [9] shows that a large part of the respondents expresses a favorable opinion regarding the development of the state CCS support policy. Despite the absence of explicit public support, the Chinese government plans to develop new large CCS projects.

Australia is one of the world leaders in the development and promotion of CCS technologies [141]. This resulted from the governmental support and other stakeholders’ interest in the implementation of the projects, especially those involving integration with the oil industry [142].

Assessment of the expert opinion on the CCS perception in Spain carried out in [40] showed that this technology is favorably perceived an intermediate step in solving the global warming issues, regardless of a slight concern about the reliability of storage and costs of capture. The experience of CIUDEN's CCS project in Spain is one of the good examples of a proactive stakeholder policy accompanied by interaction with the local community. The paper [143] formulates a number of key factors, which enabled to achieve a high level of public support, for example, a highly qualified team, community engagement plan, identification of the local community needs, etc. In general, we can say that all these factors constitute a part of a detailed project implementation plan that takes into account the diverse needs of the local community.

In contrast to successful examples, there are a number of countries, where CCS has uncertain or minimal prospects due to the difference between the stakeholders’ opinions. For example, in Finland, there is no a strong opposition against CCS, but there is no public support either as some stakeholders are not interested in the technologies application due to questionable indicators of their financial efficiency [72]. Additionally, there is no adequate regulatory framework that could increase the attractiveness of CCS technologies [144], which have certain prospects for country development.

In Sweden and Denmark, the governmental authorities demonstrate considerable uncertainty towards these issues, and public perception remains poorly studied. However, both countries have a certain potential for the implementation of the CCS process stages [135, 145].

The same situation is observed in Scotland, where the main CCS stakeholders are skeptical about the CO2-EOR projects, as unattractive and temporary [146]. At the same time, the Scottish people are loyal to almost any kind of activity [18], which is also observed in Romania, where, according to [39], even after a provision of negative information about the CCS, the technology perception has improved significantly.

In Poland, the local public demonstrates a positive reaction to the CCS projects, and expects a positive effect from their implementation, both for people and for their region in general [41, 107], despite some skepticism about the location of CO2 storages on their territory due to certain risks of leakage. But, despite the public approval, CCS projects in Poland are not implemented, as there is no effective governmental support for this process.

The prospects for the CCS development in Vietnam are connected with the necessity to create financial incentives for the technologies implementation, for example, preferential taxation for land use, development of an integrated environmental policy, and availability of international cooperation and support from the countries experienced in the implementation of such projects [147].

In France, CCS support is insignificant [148], although there is no apparent confrontation against this technology. We would rather say that people are suspicious of CCS. This results from insufficient knowledge about separate process stages, and a great number of risks.

In the Netherlands, after the cancellation of the Shell Barendrecht project in 2010, which faced apparent and unexpected public confrontation, the CCS perception can be described as negative. Besides, the national government is pursuing an active policy aimed to restrain projects of geological CO2 storage.

In Japan, the CCS perception is, to a certain extent, related to the experience of nuclear power use. Recent catastrophes have strengthened the negative perception of CCS, and increased the attractiveness of renewable energy for the local public, as well as affected the perception of CCS projects. The public opinion about the prospects of on-shore and off-shore CO2 storage became more negative due to possible leakages caused by earthquakes; although the CCS ideas themselves do not produce a negative reaction [66].

Among the countries, where CCS has faced apparent confrontation from the majority of stakeholders, including the public, Germany has the most noticeable experience. Such experience is described in many publications, which show that the German socio-economic and political situation itself is unfavorable for the CCS implementation [149]. This situation results from a popular view that CO2 emissions can be reduced by means of alternative energy, and also from a special political position of the coal industry [72].

Some studies have also noted that large-scale development of CCS projects is economically unreasonable in the country [150]. The paper [39] also shows that whatever positive or negative information is provided to the respondents, they can change their opinion to the worse. This happened to the respondents from Germany, but not from other EU countries.

When comparing the perception of onshore and offshore CO2 storage [151], many respondents from Germany could not choose an acceptable alternative, as they have a negative attitude to CO2 storage in general. At the same time, the Germans have a rather neutral opinion about the use of pipelines for CO2 transportation; although it is necessary to take into account the needs and socio-demographic characteristics of the local public in a particular area due to the NIMBY reaction [152].

Nevertheless, the papers [125, 153, 154, 155] show that the perception of the German local community can depend on the objectives of a CCS project. Comparison of the two projects in Germany showed that when a project is implemented by a scientific institution that does not profit from the project (Ketzin), the public trust is higher than in the case when a project is implemented by an energy company (Vattenfall at Beeskow). At the same time, it is noted that in the second case, the public was not sufficiently informed about the details of the project, and did not have any opportunities to influence its implementation. It should also be noted that in Germany people are quite loyal to the technologies for the production of various products from CO2 (CCUS) [156], which makes them similar to other EU countries, for example, the UK [157].

Perhaps, there is a definite correlation between a sharply negative perception of various innovative technologies by the Germans (CCS, gene technology etc.), which have a number of uncertain risks. However, at the moment, there is no reliable confirmation of this correlation in the world scientific literature. At the same time, active resistance of the German stakeholders to the CCS projects provokes an opposite reaction in the scientific community, which can be seen in numerous publications.

Taking into account different status of the CCS projects in different countries [158], we believe that an important factor of their further development is international cooperation, which would enable to combine efforts creating favorable conditions for the projects and adopt successful experience of other countries, for example, Australia [159]. Such cooperation can also appear to be an efficient tool for the development of the ideas for the environmental technologies introduction, and communicating them to the general public.

## 4 Summary

### 4.1 General review

Almost all of the reviewed articles consider two or more of the above-mentioned factors, except 3 articles [3, 45, 69], that consider only one factor. Table 6 shows the frequency of combinations of two factors: green (40 times and more), yellow (20–40 times), and red (less than 20 times). The smallest number of pairs is observed with WTP. However, this is not due to the isolation of this factor, but to a small number of articles in this area (Table 2).

**Table 6:** The frequency of simultaneous presence of two factors in one article^∗^.

	Aw	Kn	NIMBY	BR	SD	Will	Tr	Acc
Aw								
Kn	39							
NIMBY	17	27						
BR	29	55	32					
SD	15	30	14	30				
Will	6	7	5	9	2			
Tr	13	30	19	37	25	5		
Acc	31	61	24	50	25	11	27	
GovS	24	43	24	46	31	9	38	44

^∗^Aw – Awareness, Kn – Knowledge, BR – Benefits and Risks Perception, SD – Socio-demographic factors, Will – Willingness to pay, Tr – Trust, Acc – Acceptance of CCS and preference between technologies, GovS – Governmental Policy and Interaction between Stakeholders.

All reviewed articles could be divided into Qualitative (47 articles), Quantitative (83 articles) and studies with combined analysis (5 articles). The most commonly used data collection methods (Table 7) in these articles are online surveys (including one online focus group [62]), interviews and organization of various sessions (mostly focus group discussions – 14 articles).

**Table 7:** Methods of data collection distribution.

Method of data collection	Type of analysis			Total
	Qualitative	Quantitative	Combined	
Survey (not specified or traditional paper-and-pencil questionnaire)	1	27	1	29
Mail survey	0	7	0	7
Information-choice questionnaire	2	11	0	13
Online survey	2	28	1	31
Interviews	17	17	2	36
Telephone survey	3	8	0	11
Media analysis	4	3	0	7
Various sessions (workshops, seminars, panels, focus group)	12	15	3	30
Theoretical (including reviews and case studies)	22	0	0	22

The most common research methodologies (Table 8) are descriptive statistics and various types of parametric analysis (mostly regression analysis – 33 studies). Only in three studies [69, 150, 163], modeling elements based on ecological-economic indicators are used. It should be noted that the TPB (Theory of Planned Behavior) is a base for a significant number of Quantitative studies related to the influence of various factors on public perception. However, this fact clearly stated only in 11 articles.

**Table 8:** Methodologies distribution.

Methodology	Type of analysis			Number of studies
	Qualitative	Quantitative	Combined	
Case study	10	5	1	16
Review	5	0	0	5
Ecology-economical modeling	0	3	0	3
PESTEL analysis	0	1	0	1
Non-parametric analyses (Wilcoxon tests, Mann-Whitney U tests, Kruskal-Wallis tests, chi-squared test, Friedman test, component, and structure analysis)	0	13	0	13
Descriptive statistics (frequencies, means, standard deviations, correlations)	0	68	4	72
Parametric analyses (t-test, ANOVA, regression analysis, cluster analysis)	0	44	2	46

**Figure 2:**
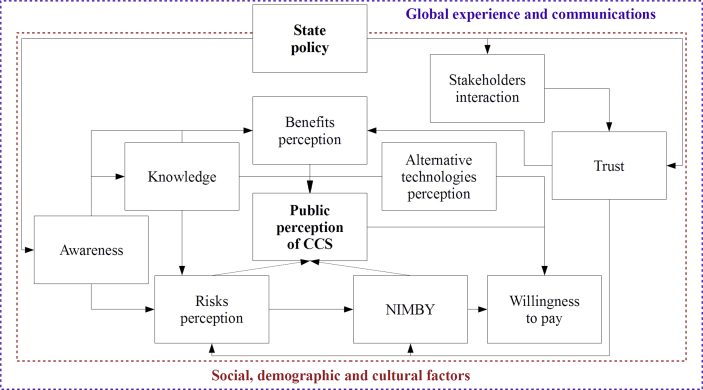
Main linkages between studied groups of factors and public perception of CCS.

As a result of the analysis, a scheme (Figure 2) reflecting the main linkages between the considered groups of factors and their relationship with the public perception of CCS was drawn up. The following subsections discuss the findings for each of the mentioned groups.

### 4.2 Awareness

The approach to public awareness raising should be based on a detailed preliminary analysis, which will enable to identify specific socio-economic characteristics of the public, their motives, and factors that can arouse interest in new knowledge. The studies define public awareness as one of the key factors providing an objective CCS perception and determining the degree of understanding of the technology. Nevertheless, the existing scientific background does not enable to determine the exact scope of information about CCS that would be exhaustive. This is important, as both insufficient and excessive information can lead to misunderstanding of the CCS fundamental principles.

In addition, a certain misinterpretation of the facts, which some project stakeholders may be interested in, can appear a more efficient tool for them. In this case, the public can be misled, which will enable to achieve some short-term goals, but, in the long term, this can lead to open protests. To avoid potential misinterpretation of the facts, we consider it necessary to involve stakeholders who are independent from each other, and who will not allow a distortion of the real picture.

### 4.3 Knowledge

Promotion of the public understanding in the field of sustainable development, including global warming and the technologies used to fight it (for example, CCS), has a dual character. On the one hand, it enables involve more people in an open discussion, and, therefore, review potential risks and effects of CCS more thoroughly. On the other hand, this enables us to launch reframing of an environmentally balanced society development, which will include not only assessment of industrial environmental projects, but also an understanding of the individual responsibility of every person.

At the same time, it is necessary to have a clear idea of the sources, where the public knowledge originates from. If people do not have an opportunity to rely on expert opinion about the quality of a material, they will turn to the Internet, where information can be inconsistent and unreliable. As a result, during the project implementation, the stakeholders will have to fight against false judgments, rather than develop the required public knowledge, which seems to be a much more labor-intensive activity.

When we talk about a specific project and the local public, the lack of reliable knowledge about CCS can become, on the one hand, an instrument of opposition in the fight against the project. On the other hand, it will allow unscrupulous stakeholders to deceive the local public in relation to key aspects of the project. Thus, dissemination of knowledge about the nature of technology among the local public is necessary to prevent possible conflicts.

### 4.4 Not in/under my back yard

The NIMBY effect is a natural reaction of a person to the unknown, in particular, to unfamiliar technologies such as CCS, which still remain poorly studied, especially, in the area of geological carbon dioxide storage. There are no methods eliminating this effect completely; however, it is possible to mitigate the negative perception of lay people, provided that it is identified in the early pre-project stages. This necessity is related to the explicit preferences of the local public for various process solutions at all stages of the CCS production chain. In addition, the NIMBY reaction can be reduced by provision of the most complete information about the measures taken to ensure the safety of the local public, as well as the development of measures aimed to stimulate the local public.

### 4.5 Benefits and risks perception

It is the risks and benefits perception that underlies approval/disapproval of the CCS technology. At the same time, the perception of technology is more influenced by the perception of the benefits, whereas the perception of the risks is an indicator of a protest potential. This means that it is necessary to develop a single and considered policy governing all stakeholders’ interaction with the public, which will allow to equally influence both of these factors.

All risks and benefits discussed in the reviewed articles can be divided into five groups (Table 9). With regard to the benefits of technology, the environmental benefits of reducing greenhouse gas emissions are most often mentioned. In addition, a large number of articles highlight the benefits for society and for the project area (44 studies), such as job creation, the attraction of investments, etc. The study of risks is more common than benefits, due to the lack of knowledge about the technology and the lack of intrinsic public knowledge about its essence. At the same time, 70 articles refer to risks as consequences of CCS implementation but do not specify their nature.

**Table 9:** Mentions of risks by groups.

Risks	Number of studies	Benefits	Number of studies
Risk for the society	40	Benefits for the society	44
Risk for personal safety	42	Benefits for oneself	17
Risk for the environment	51	Benefits for the environment	53
Risk for future generations/long-term sustainability	16	For future generations	9
General risks	70	General benefits	34

Among the specific CCS risks (Table 10), the most frequently mentioned is the risk of CO2 leakage. The second most frequently mentioned risk is the risk of failure to implement plans to reduce CO2 emissions. This risk includes one dual point – support for fossil fuels. This point is perceived both in terms of risks (continued use of environmentally harmful fuels) and in terms of benefits (the possibility of using cheaper energy without a significant impact on the environment). The least mentioned risks are usually considered in articles on the perception of CCS by local residents of the regions, where the projects are planned to be implemented.

**Table 10:** Most mentioned risks.

Risks	Number of studies
CO2 leakage, migration (from storage or pipeline) or explosion	48
Disposal of CO2 may cause seismic activity	29
Environmental impact (underground, marine environment)	31
CCS cannot achieve the goals of reducing CO2 emissions because of the lack of effectiveness and lack of facilities for storage. This is just a temporary solution that supports the use of fossil fuels.	34
Possible destruction of facilities due to the lack of stakeholders' responsibility.	9
Loss of land due to the construction of infrastructure.	9

Despite the results of this review, we should bear in mind that the expected benefits and the most important risks can differ by countries, regions, cities and even social classes. A certain impact on the risks and benefits perception can be ensured by a proper presentation of the technology, which will provide the public with fair information on the measures to be taken to assure its safety, and the benefits for the regions, where the projects will be implemented.

### 4.6 Socio-demographic factors

Socio-demographic factors determine a worldview of a person, his position in the context of global problems, understanding of the importance of individual responsibility for environmental protection. Most of these factors cannot be controlled and managed, and therefore, other CCS project stakeholders have to adapt their work methods to a specific target audience. At the same time, in most cases, a detailed study of its characteristics enables to determine potentially efficient options of interaction between stakeholders.

To prepare the acceptable methods of community engagement, we need to collect a considerable amount of information to characterize the target audience. We cannot prefer one information collection method over another, as the local public can include people of different ages, confessions, and worldviews. Nevertheless, the involvement of people, whom the local public trusts, can have a certain contribution to the collection of the necessary amount of reliable information. It is necessary to keep in mind that collection of socio-demographic data about the target audience is a normal pre-interaction stage, because other stakeholders need to know, who will be their partner in the project.

### 4.7 Willingness to pay for CCS

In the coming decades, the growth of energy rates is an inevitable trend, both in case of renewable energy development, and expansion of fossil fuels use with or without CCS. Nevertheless, the willingness to support technology that will accelerate the growth of energy rates is a significant barrier to large-scale implementation of CCS. This problem already exists in countries aware of CCS and is expected in those countries, where CCS is only at the initial stage of development.

Some studies show that a positive perception of the technology enables rising energy rates for the public on a voluntary basis; however, such growth is extremely limited and only possible in a few countries. In addition, the willingness to pay for the technology implementation is closely related to the quality of life; therefore, it is necessary not only to increase the technology attractiveness but also develop adequate socio-economic incentives.

### 4.8 Trust

It seems that trust is a central element determining a positive perception of CCS technologies. It is the public trust in stakeholders that determined their readiness to consider a project implementation option. This can be observed even in the experience of Germany, where the CCS ideas themselves cause a negative public reaction. At the same time, earning of public trust is an extremely long and labor-intensive process, which largely depends on the experience of the interaction between lay people and the project stakeholders. In addition, in the case of the negative experience, it will be difficult to change the public opinion for the better. For example, such a situation exists in the relationships between the local public in some regions of Russia and local governmental authorities.

### 4.9 Acceptance and preferences between technologies

Objectively, in most countries, the CCS projects are less preferable as compared with renewable energy. However, the history of renewable energy development shows that its large-scale implementation started only as a result of balanced and aggressive marketing policy, and huge state support in a number of countries. As for CCS, in a number of studies, the respondents note that such supporting factors are not provided for these technologies. We can say that in countries where the ideas of a positive impact of the renewable energy development, confirmed by a number of successful projects, has been already established; CCS is perceived as a competing technology, and its support is equivalent to a delay in the renewable energy development.

However, it should be noted that the renewables cannot completely replace fossil fuels in the near future, given the current industry growth rates. This fact is rarely mentioned in the information materials for lay people. Additionally, CCS is not a direct competitor to renewable energy, as the purpose of this technology is to increase the environmental safety of fossil fuels, rather than replace them. Thus, on the one hand, it is necessary to eliminate the public misconception opposing these technologies. On the other hand, we should show the spheres of influence and contribution of each technology in the environmentally sustainable development of the society.

### 4.10 Governmental Policy and Interaction between stakeholders

The state environmental policy plays a defining role in the efficiency of further CCS development, as any innovative technology at the stage of its development needs substantial support. The experience of some countries shows that CCS can be actively implemented only with a long-term development strategy, which, among other things, determines a stakeholders’ engagement procedure and their responsibilities.

For the public, successful implementation of the CCS projects largely depends on a well-coordinated work of all stakeholders with due consideration of their opinions. In addition, the arguments for lay people in favor of the CCS project implementation, which can cover ecological, technical, or economic issues, should be expressed by those stakeholders, whose goals the public approves, and whose opinion the public trusts.

### 4.11 Cross-country outlook

At the national level, there is a large huge number of factors that can influence the public perception of technology, its approval or willingness to protest against its implementation. At the same time, according to our review, these factors are difficult to reveal as they largely depend on the specific features of a region to be assessed, the mentality of the local public, actions of local and regional authorities, and other things. Even when several projects within the EU are assessed, we have to admit that the key factors of their performance are not exhaustive, and can have different importance when considering projects in other countries. Nevertheless, the main groups of factors remain the same: "benefits", "costs/risks", "climate change".

The necessity to identify specific factors suggests that the best starting point for promotion of the CCS technologies is to study socio-economic characteristics of the target audience, their views, and knowledge about the role of environmental projects, for example, by means of online surveys that showed good results in the international practice. This initial step will allow to find ways of achieving the balance of interests, which should be further discussed during dialogues and various sessions. On the one hand, it will enable to determine the necessary measures increasing a general level of awareness; on the other hand, it will enable to demonstrate the authorities’ interest in the public opinion, and, consequently, improve the public trust in one of the key CCS stakeholders at the initial stage — the state. For example, one can use elements of the approaches to the Social Site Characterization proposed in [160] (main principles of public participation), and [161] (approach to the CCS project management).

## 5 Conclusion and further research

The analysis showed that the public is an important stakeholder of CCS projects, the opinion and needs of which should be taken into account. In this regard, it is necessary to improve the mechanism of interaction between stakeholders in order to find compromises at all stages of the project, starting with the planning of the site selection. Such improvements require an interdisciplinary approach and the identification of key goals for further development of research in this area. As such goals, it is possible to set the necessity of overcoming key barriers for the CCS implementation, which were identified in this review (Table 11).

**Table 11:** Key barriers for CCS implementation.

Barriers for CCS implementation	Number of studies
Lack of public knowledge about CCS, misconceptions.	95
Lack of or poor communication strategy	82
Competition between alternative technologies	70
Lack of long-term policy of CCS implementation	57
Controversial economic efficiency, capital-intensity, weak market-based mechanism	55
Not enough studied the long-term effects of the technology	52
Lack of trust in some stakeholders	54
NIMBY reaction	38
Site selection and project design without taking into account the specific of locals	34
Appearance of protest potential due to negative public perception	25
Increase in price of energy	22

To overcome these barriers, it is necessary not only to find new ways of cooperation between stakeholders, but also to improve our knowledge about the possible consequences of this technology, to provide this new knowledge to the public, to create suitable economic conditions and to determine the necessity of CCS projects by comparison with other low-carbon alternatives. At this step, it is necessary to mention a significant gap in our knowledge about alternative options of CO2 sequestration, such as CCUS and CCU, which also should be taken into account during the assessment of alternatives. The differences between these options should be widely discussed and investigated, because they have different CO2 management principles and organizational features [162]. These differences could also have a significant influence on a public perception of different CO2 sequestration options.

In terms of the geographical distribution of CCS public perception research, this review will form the basis of the first studies in the field of the CCS technologies promotion in Russia. Therefore, preparation of a sound plan for the development and promotion of the CCS projects is a relevant and up-to-date objective.

A positive feature of the Russian CCS projects development is the presence of a significant number of depleted oil fields located far from residential areas, which can be used for CO2-EOR. Production of additional oil volumes can have a positive effect on public perception [65]. In addition, a preliminary assessment of the CCS-EOR projects financial performance in Russia showed that they can be economically efficient under the current conditions [163].

However, while the possibility of national companies’ efficiency increase will be positively perceived by the public, this is not obvious for an environmental component of such projects. Usually, environmental pollution is a local problem of the regions; therefore, the same opinion can also be expressed regarding greenhouse gas emissions [164]. In particular, this situation is typical for Russia, which occupies a huge territory, where people are rather poorly informed about the events occurring at the other end of the country and even in neighboring regions [165].

In addition, Russia needs to develop a compensation mechanism for the public and other stakeholders' risks at the expense of excess revenues from oil production; as the companies do not consider such risky and long-term projects, and they require state support in the form of socio-economic mechanisms that require co-financing, insurance and/or implementation of a risk management system for the projects [147]. For this purpose, it is necessary to develop appropriate legislation in the field of CCS, as well as a general environmental policy [166] defining the responsibilities of each stakeholder. These measures will enable to protect the stakeholders' interests, and ensure financial security. The importance of such measures is determined by a dual nature of stakeholders (industry and NGOs) in the CCS projects. Firstly, they are interested in the project's implementation from their professional point of view. Secondly, they are the most competent experts in their disciplines, which can have a positive effect on the CCS public perception in Russia [35].

## Declarations

### Author contribution statement

All authors listed have significantly contributed to the development and the writing of this article.

### Funding statement

The research was carried out within research school «Rational subsoil use» in the Department of Organization and Management; with the financial support of the grant of the Russian Science Foundation (Project No. 18-18-00210, «Development of assessment methodology of public efficiency of projects devoted to carbon dioxide sequestration»). Saint-Petersburg Mining University.

### Competing interest statement

The authors declare no conflict of interest.

### Additional information

No additional information is available for this paper.

## Figures and Tables

**Table:** 

The first author/author	Year	Sample (number of respondents)	Level*	Focus	Method of data collection**	Aim of the research
Anderson C. [120]	2012	20	Local	Project experience, public acceptance	Interviews, telephone survey	to explain community acceptance of CCS through a human and social capital analysis, and through that analysis assess the Otway public participation process.
Anghel S. [46]	2017	1002	General	Public perception, communication	ICQ	to collect and analyze data on Romanian public awareness and knowledge of climate change, energy policy and CCS in general and furthermore on public awareness and knowledge about local demo initiatives and existing CCS information material and campaigns.
Arning K. [156]	2017	232	General	Public perception, risk perception	Focus groups, online survey	to explore public perception of carbon dioxide utilization technologies in Germany; to conceptualize carbon dioxide utilization risk perception and acceptance.
Ashworth P. [114]	2009	41	Local	Public perception	Workshops	to understand public perception to climate change and low-emission technologies and how to engage communities on these topics.
Ashworth P. [31]	2009	305	General	Effectiveness of large group dialogues	Workshops	to explore Australian society's acceptance of energy technologies; to assess the effectiveness of dialogue with large groups for informing knowledge and changing attitudes of low emission energy technologies.
Ashworth P. [71]	2015	N/A	General	Public communications	Qualitative, literature review	to collect results related to public communication on CCS
Ashworth P. [105]	2012	N/A	Local, cross-country	Comparison of projects experience	Interviews, telephone survey	to identify factors that contributed to successful project deployment, as well as to assess lessons learned about various communication and engagement practices.
Ashworth P. [99]	2009	N/A	General	Communication activities	Qualitative, online survey, telephone survey	to synthesize the range of communication activities that have been planned or implemented since 2002 in Australia and internationally, and examines the strengths and weaknesses of these activities.
Ashworth P. [18]	2013	374	Cross-country	Public perception	Workshops, questionnaire	to explore how international context may have impacted on the results of engaging the general public on issues related to climate change, energy technologies, and the overall shift towards a low carbon society; to discuss arised differences and the implications for policy makers and research developers.
Billson M. [128]	2017	N/A	Cross-country	Policy	Qualitative	to show the relevance of state support for CCS projects implementation
Boyd A.D. [139]	2017	1471	General	Public perception, EOR	Online and telephone survey	to examine descriptive statistics to understand public perceptions of CCS and applied regression models to assess how risk perceptions, perspectives of climate change and trust in government relate to the support for or opposition to CCS development and funding for the technology.
Bradbury J. [76]	2009	N/A	General-Local	Public perception	Focus group and interviews	to discuss findings from the joint review of the focus groups and the potential lessons for research and application to CCS deployment.
Braun C. [149]	2017	3526	General	Preferences between technologies, public perception	Online survey	to compare public perception in Germany of three specific measures: solar radiation management via stratospheric sulphate injection, large-scale afforestation, and carbon capture and sub-seabed storage.
Breukers S. [77]	2015	15	General, cross-country	Public engagement	Qualitative, interviews	to improve understanding of how project developers view and practice engagement and communication.
Brunsting S. [160]	2013	1850	Cross-country	Social site characterization	Focus conferences, paper-and-pencil questionnaire, telephone interviews, media analysis	to present the results of social site characterisation and public participation activities at two prospective CCS sites in Poland and Scotland.
Brunsting S. [94]	2011	N/A	General	Public perception, communication	Qualitative, Case study	to explore the differences among chosen case studies to develop communications exercises (or even a part of consultation policy) for future CCS projects.
Budinis S. [163]	2018	N/A	General	Prospects of CCS	Modeling	to identify and review potential CCS barriers, with a focus on CCS costs;
Buhr K. [44]	2014	N/A	General	Communication approaches, public engagement	Qualitative	to explore assumptions made about senders and receivers of information when involving the public in CCS communication and how these assumptions relate to different communication objectives.
Carley S.R. [47]	2012	1001	Local	Public perception	Telephone survey, mail survey, interviews	to examine early public impressions of CCS in a coal-intensive state, Indiana.
Chaudhry R. [126]	2013	84	General	Energy policy, stakeholders' perception	Qualitative, Interviews	to assess variation in the state-level energy context for CCS development by exploring energy policy stakeholders' perceptions of CCS in four geographically and demographically diverse states.
Chen Z.-A. [9]	2014	679	General	Public perception	Questionnaire	to assess public understanding of the climate sciences, society's knowledge and acceptance of low emission technologies, public interests and concerns about the positive and negative impacts of CCS technology, and public attitudes towards CCS policies supported by the government.
Cherry T. L. [111]	2014	674	General	Public perception, opposition	Telephone interviews	to provide insights to the origins of public opposition that can impede the adoption of low-carbon technologies by investigating how perceptions are shaped by local economic interests and individual cultural worldviews.
de Best-Waldhober M. [119]	2012	971	General	Preferences between technologies	Mail survey, ICQ	to measure informed opinions regard- ing CCS in comparison with other CO2 emission reduction options by combining valid and well-balanced information with a large sample that is representative of the Dutch public.
de Best-Waldhober M. [113]	2008	1322	General	Public perception, acceptance	Information Choice Questionnaire	to analyze the awareness and perception of the Dutch general public regarding CCS.
de Best-Waldhober M. [33]	2012	846	General	Public perception	Questionnaire, interviews, media analysis	to enhance insight into currently held beliefs and awareness among the general public about CCS; to investigate the role of the media as a vehicle for knowledge transfer.
de Bruin W.B. [45]	2014	891	General	Public perception, impression formation	Online survey	to learn more about how people respond to a validated educational communication about CCS.
De Coninck H. [141]	2009	N/A	General	International cooperation	Qualitative	to show the relevance of strong international cooperation on CCS demonstration.
Desbarats J. [125]	2010	N/A	General	Review	Qualitative, Case study	to describe the results of the European project NearCO2 first phase, which focuses on lessons learned from CCS and analogous developments in recent years.
Dowd A.-M. [38]	2014	2470	Cross-country	Public perception, knowledge	Online survey	to address the gap around identifying what is the public's knowledge of CO2 properties, sources, uses and effects; to examine the relationship of existing knowledge on perceptions of CO2 and CCS; to explore the effect of information provision on knowledge and opinions of both CO2 and CCS.
Duan H. [87]	2010	534	General	Awareness, attitudes towards technology, determinants of acceptance	Mail survey, online survey, interviews	to explore the public's perspectives on the development of CCS in China.
Duetschke E. [53]	2014	1830	General	Public perception, preferences between CCS system elements	Online survey	to investigate the relevance of different specifications of the three main steps of CCS on the public perception of CCS as well as possible interactional effects between the specifications.
Duetschke E. [155].	2011	13	Local	Comparison of projects experience	Qualitative, Case study, Interviews	to analyze and compare projects' properties, communication strategies, public perception, local context and history; to identify factors that contributed to the respective positive or negative reaction of the local public.
Duetschke E. [37]	2016	1830	General	Public perception, preferences between CCS system elements	Online survey	to examine the public perception of CCS in more detail by looking into different options within the CCS chain, i.e. for the three elements capture, transport and storage.
Einsiedel E.F. [84]	2012	82	Local	Public deliberations	Workshop, questionnaire	to examine citizens' views on climate change and a number of energy systems, with a specific focus on the use of carbon capture and storage (CCS) as a technology to address greenhouse gas emissions.
Fischedick M. [4]	2009	232	General	Public acceptance, stakeholders' opinion	Media analysis, interviews	to understand the relevance of technical and non-technical aspects of CCS in terms of social acceptance.
Fleishman L.A. [112]	2010	60	General	Preferences between technologies	Workshops, ICQ	to examine people's informed decisions about electricity-generating technologies.
Gough C. [132]	2014	19	Local	Public perception of CO2 pipeline	Focus group	to assess individuals' understanding of CO2 and identify their existing perceptions of it; to explore perceptions of risk and key areas of concern with respect to pipeline transportation of CO2 for the purposes of CCS.
Gough C. [127]	2010	31	General	Prospects of CCS in UK	Qualitative, workshop	to present the results of the workshop aimed at formulation of CCS long-term roadmap in UK taking into account opinion of wide range of stakeholders.
Gough C. [36]	2017	Around 32 (incl. 10 stakeholders)	General	Social license	Workshops, interviews, media analysis	to summarise results from empirical research with the broad aim of exploring societal responses to CO2 storage, framed around the concept of social license to operate.
Gough C. [95]	2018	12 interviews	General	Public perception	Focus group, interviews	to explore the social context for CO2 storage in the UK; to assess potential social responses to subsurface injection and site monitoring approaches; to identify significant factors in establishing a social license in the context of CCS and in particular offshore CO2 storage in the UK.
Gough C. [67]	2002	19	General	Public perception	Focus group	to explore public reaction about burying CO2 under the sea.
Ha-Duong M. [148]	2009	1076	General	Public perception	ICQ	to explore awareness about CCS in France, and the degree of approval of or opposition to the idea in the general population; to explore the variability of this opinion relative to the provision of information; to explore the variability of this opinion relative to the semantics used to describe the technology.
Hansson A. [145]	2005	12	General	Stakeholders' perception	Interviews	to examine the attitudes of Swedish politicians, scientists, NGOs and industry regarding CCS, i.e. actors who possess knowledge about CCS today and will influence the public opinion of tomorrow.
Haug J.K. [135]	2016	N/A	Cross-country	Local acceptance	Qualitative	to assess the Nordic situation with regard to carbon capture and storage (CCS) deployment at the local level.
Hope A.L.B. [82]	2014	20	General	Role of religion in attitude towards CCS	Focus groups, questionnaire	to explore potential differences between the Muslim, Christian and secular participants in terms of pro-environmental values and beliefs; to explore how attitudes to CCS and climate change were shaped by religious beliefs.
Howell R. [24]	2013	99	General	Public perception	Workshop	to present the results of a large group process conducted in Edinburgh, Scotland investigating public perceptions of climate change and low-carbon energy technologies, specifically CCS.
Itaoka K. [34]	2009	2490	General	Public perception	Online survey	to investigate the extent of recognition and latent social acceptance on global warming mitigation measures including CCS, as well as the kind of factors that would influence their views.
Itaoka K. [91]	2016	548	General	Public preferences between low-carbon technologies	Door-to-door survey	to examine the rationale for policy parity of basic low carbon power sources and the consumer preference for those power sources.
Itaoka K. [66]	2014	1251	General	Dependence between public perception and natural, and technogenic accidents	Online survey	to measure the influence of the large earthquakes and nuclear plant accidents on public perception of CCS.
Johnsson F. [104]	2009	142	Cross-country	Stakeholder perceptions	Questionnaire	to identify, study, and address non-technical issues associated with CCS from fossil-fired plants in the energy sector, and to provide guidance to decision makers.
Jones C.R. [121]	2014	16	General-Local	Public perception of CO2 utilization technologies	Focus group, ICQ	to design and test a methodology for investigating public perceptions of CDU; to elucidate new understanding of people's attitudes towards the technology.
Jouvet P.-A. [69]	2014	0	General	Social acceptance, balance between tax and pollutions	Modeling	to determine, from the social point of view, simultaneously the amount of production as well as the optimal allocation of CO2 emissions between the atmosphere and underground storage sites.
Kaiser M. [107]	2014	1006	General	Public engagement, public perception	Interviews, media analysis, focus group	to analyze the local public perception of CCS among citizens and stakeholders; to inform community representatives and the local public about CCS technology and to involve them in the planning process for the prospective CCS project.
Karayannis V. [92]	2014	N/A	General	Public perception, economic aspects	Qualitative	to discuss recent socio-economic aspects of CCS technologies.
Karimi F. [3]	2017	19	Cross-country	CCS policy	Interviews	to define temporal features (i.e. frame, timing, tempo, and duration) for policy making and deployment of large-scale CCS projects.
Karimi F. [68]	2018	13901	Cross-country	Public perception, cross-cultural differences	Based on Eurobarometer	to explain the importance and role of cross-cultural differences and the reaction of people in different countries towards the technology vis-à-vis the other factors and demonstrate how those differences operate.
Karimi F. [72]	2015	19	General	Experts' risk perception	Interviews, Case study	to contribute to the risk governance of CCS by investigating the concerns of experts about CCS and the role of socio-cultural factors in their risk perception.
Karimi F. [81]	2016	13901	Cross-country	Influence of socio-cultural factors on risks and benefits perception	Based on Eurobarometer	to explore how do cultural structures of a society affect benefit and risk perception of CCS and what extent is the reaction of the public to implementation of the technology predictable in a crosscultural comparative framework.
Karimi F. [73]	2014	13901	Cross-country	Relation between cultural factors and risks perception	Based on Eurobarometer	to explore how do cultural structures of a society affect risk perception of CCS and what extent is the reaction of the public to implementation of the technology predictable.
Klass A.B. [124]	2008	0	General	Liability	Qualitative	to create a potential framework to address liability and funding issues associated with the long-term storage of CO2 in connection with CCS.
Kraeusel J. [89]	2012	130	General	Public acceptance, willingness to pay	Online survey	to explore social acceptance and willingness to pay for the Carbon Capture and Storage technology in Germany.
Krause R.M. [54]	2014	1001	General	Public perception, NIMBY	Telephone–mail–telephone survey	to examine how the closeness of a hypothetical CCS facility to individuals' communities influences their acceptance of it.
Kubota H. [19]	2017	23612	General	Public perception	Online survey	to analyze the attitudes and perception of CCS and thermal power generation through internet questionnaire surveys, and to provide appropriate information to promote public understanding and decision-making for introducing CCS technology for thermal power plants.
Li Q. [118]	2014	679	General	Public perception	Questionnaire	to explore public perception of CCUS in China.
Li Q. [28]	2017	570	General	Public perception	Questionnaire	to investigate the public awareness and understanding of the environmental impact and management of CCUS technology.
Lupion M. [143]	2013	N/A	Local	Project experience	Qualitative, Case study	to describe the integral communication plan and public outreach strategy designed and implemented in the areas of influence of CIUDEN's large facilities on CCS.
Mabon L. [26]	2013	23	General	Public perception, offshore storage	Qualitative, Interviews	to challenge arguments that, due to the greater distances from centres of population, it will be ‘easier’ to garner public and stakeholder support for offshore CO2 storage than onshore.
Mabon L. [86]	2013	72	Cross-country	Public perception	Qualitative, Interviews	to illustrate how publics and stakeholders often evaluate the geological storage of carbon dioxide in terms of its relation to their broader world views, rather than purely in terms of the perceived techno-scientific risks of the technology.
Mabon L. [146]	2015	N/A	General	Stakeholders' perception, policy	Focus groups	to overview the key aspects of CO2-EOR stakeholders interaction and perception of such projects.
Malone E.L. [42]	2010	N/A	General	Stakeholders involvement	Qualitative	to discuss the issues involved in providing information as part of the CCS survey, maintaining that such information is never unbiased and thus tends to produce pseudo opinions that reflect the pollster's or researcher's bias.
Markusson N. [2]	2012	N/A	General	Social dynamics of technology	Qualitative	to explore the role of social sciences in the development of CCS
Midden C.J.H. [49]	2009	112	Local	Trust, risk perception	ICQ	to analyze the role of trust and risk perception in attitudes formation towards CO2 storage.
Miller E. [50]	2007	1273	General	Socio-demographic differences, trust, knowledge	Online survey	to explore the extent to which socio-demographic characteristics influence knowledge, trust, risk perception and acceptance of CCS.
Miller E. [51]	2008	1273	General	Public perception	Online survey	to provide a benchmark of perceptions and initial reactions to geosequestration technologies in Australia; to explore some principles for the development of effective engagement and risk communication strategies that appropriately address the specific needs and concerns of the Australian public.
Moutenet J.-P. [43]	2012	2616	Local	Public acceptance	Online survey	to get information about public acceptance for a potential CCS pilot project in the province of Quebec.
Nicole M.A. [108]	2007	103	Local	Public perception	Questionnaire	to analyze public judgments of the acceptability of CCS, in particular how these evolve and get shaped in the social context comprising of the professionally involved actors, and how opinion formation of lay citizens and that of professionally involved actors interact.
Offermann-van Heek J. [122]	2018	137	General	Trust, public perception	Interviews, online survey	to investigate the connection between trust in CCU companies and the acceptance of innovative CCU products.
Oltra C. [153]	2012	51–69	Local, cross-country	Comparison of projects experience	Qualitative, case study	to examine the development of public reactions in relation to five European CO2 storage projects; to identify any lessons that may be learned for the future.
Oltra C. [20]	2012	500	General	Public perception	Online survey	to analyse how additional information on CCS affects individuals' reactions to CCS.
Oltra C. [16]	2010	52	General	Public perception	Focus group	to analyze the lay understandings and perceptions of CCS technologies and projects in Spain.
Palmgren C. [137]	2004	144	General	Public perception	Questionnaire, interviews	to explore likely public perceptions in the United States of CO2 disposal in deep rock formations and the ocean.
Perdan S. [157]	2017	1213	General	Public perception, awareness	Online questionnaire	to establish the extent of people's awareness and acceptance of CCUS and to elicit the importance they put on different sustainability issues relevant to CCUS.
Pietzner K. [39]	2011	6168	Cross-country	Public perception, awareness	Questionnaire	to summarise the results of public perception and awareness surveys in six European countries - Germany, Greece, the Netherlands, Norway, Romania and the United Kingdom (UK).
Pihkola H. [144]	2017	0	General	Sustainability of CCS	Qualitative	to discuss the sustainability of CCS technologies from a cross-disciplinary point of view.
Prangnell M. [140]	2013	N/A	General	Public communications	Qualitative, Case study	to describe key aspects of CCS image crisis.
Reiner D. [115]	2006	4009	Cross-country	Public perception	Paper-and-pencil survey, online survey	to compare public attitudes in the United States, United Kingdom, Sweden and Japan towards key questions of energy and the environment, with particular emphasis on attitudes towards carbon capture and storage (CCS).
Reiner D.M. [116]	2006	4009	Cross-country	Preferences between technologies	Online survey, questionnaire, telephone survey	to explore difference in CCS perception, compared to other low carbon technologies, between countries.
Riesch H. [62]	2013	942	Cross-country	Public perception	Online focus group discussion	to discuss online focus groups as a deliberative method in experimental and perhaps consultative contexts; to show the role of anchoring and associative reasoning in the development of public opinion of CCS; to discuss the managing public-facing energy messaging in an age of public access to online information.
Rychlicki S. [41]	2015	90	General	Public perception, social acceptance	Questionnaire	to explore public sentiment associated with using CCS and CO2-EOR technologies in Poland.
Sacuta N. [106]	2017	0	General	Projects experience	Qualitative, Case study	to examine the public outreach enacted for three different CO2 injection projects to identify differences and similarities in the strategies employed for public dissemination of information.
Sala R. [40]	2011	97	General	Stakeholders' perception, social acceptance	Online survey	to report an empirical analysis of stakeholder perceptions on the risks, challenges and barriers facing CCS deployment in Spain.
Schumann D. [70]	2012	N/A	General	Public acceptance	Qualitative	to overview methods of CCS acceptance research.
Schumann D. [152]	2017	1000	General	Public perception, pipelines	Computer-aided telephone interviews	to investigate the public perception of CO2 pipelines among the German public.
Schumann D. [151]	2014	2003	General	Public perception	Interviews	to investigate and compare the public perception of CO2 offshore storage, CO2 onshore storage and CO2 transport via pipeline in Germany nationwide and in two coastal regions.
Seigo S.L. [29]	2013	30	General	The role of illustrations	Interviews	to take a closer look at what constitutes a good illustration of CCS and how illustrations can impact perception of the technology.
Seigo S.L. [57]	2014	1510	General-Local	Risk and benefit perception	Online survey	to explore if there are differences in terms of risk and benefit perceptions of CCS between regions with different stages of CCS deployment.
Seigo S.L. [75]	2011	200	General	Public perception, communication	Online survey	to investigate the influence of information about monitoring measures at CO2 storage sites on laypeople's perceptions of CCS.
Selma L. [5]	2014	N/A	General	Review	Qualitative, Literature review	to review and analyze public perception research.
Shackley S. [22]	2005	212	General	Public perception	Panel discussion	to explore public perceptions of carbon dioxide capture and storage, both when first presented with the idea and when more background information is provided; to explore perceptions of the key risks and concerns surrounding CCS and what information, policies and processes would make CCS more and less acceptable to the public.
Shackley S. [88]	2008	512	Cross-country	Stakeholders' perception, public perception	Questionnaire	to analyze social acceptability on the part of both the lay public and stakeholders; to examine the acceptability of CO2 capture and geological storage within the Clean Development Mechanism of the Kyoto Protocol.
Sharma S. [142]	2006	0	General-Local	Project experience	Qualitative, Case study	to show an experience of solving a number of regulatory, organisational and social challenges which were occurred within the Otway Basin CCS Pilot Project.
Sharp J.D. [74]	2009	1972	General	Public perception of benefits and risks, level of support	Online survey	to investigate the public's perceptions of the benefits and risks of CCS, the likely determinants of public opinion, and overall support for the use of CCS.
Stephens J.C. [79]	2009	100	General	Public perception, learning	Seminar, ICQ	to explore stakeholders' perceptions of the risks and benefits of CCS technology, and how those perceptions changed with additional information provided by CCS technology experts.
Ter Mors E. [21]	2010	220	General	Stakeholders collaboration	ICQ	to examine whether people expect more balanced information from diverging collaborating stakeholders than from individual stakeholders; to examine the implications of (im)balance expectations for expected and perceived information quality; to examine whether the effectiveness of joint communications depends on the perceived (dis)similarity of the collaborating stakeholders; to assess whether occasional collaboration between stakeholders affects the perceived credibility of individual stakeholders.
Ter Mors E. [32]	2013	308	Cross-country	Research techniques, quality of public opinion	Information-choice questionnaire, focus group	to examine and compare the quality of opinions created by focus group discussions and information-choice questionnaires related to CCS.
Ter Mors E. [129]	2009	N/A	General	Public information	Qualitative, ICQ	to examine whether public information would be more effective (i.e., perceived to be of greater value) when multiple stakeholders communicate information about CCS in collaboration instead of doing so separately.
Terwel B.W. [103]	2008	393	General	Trust	Online survey	to explore how organizational motives and organizational communications affect public trust in these organizations.
Terwel B.W. [134]	2010	203	General	Group voice, trustworthiness	ICQ	to examine whether group voice affects people's perceptions of the trustworthiness of the political decision maker; to examine whether perceived trustworthiness of the political decision maker influences acceptance of people's decision.
Terwel B.W. [97]	2012	811	Local	Public perception, opposition	Telephone interviews	to determine how widespread the local resistance against the proposed CCS project actually was at this point of time (it was before Barendrecht project rejection); to determine to what extent the CCS project was an important issue for the people of Barendrecht; to explain the local public's attitudes toward the CCS project.
Terwel B.W. [101]	2010	148	General	Trust, public perception	Questionnaire	to highlight public trust in CCS stakeholders as an influential factor of public acceptance.
Terwel B.W. [55]	2012	205	General-Local	Public perception, NIMBY	Questionnaire	to determine whether the psychological structure of initial attitudes towards plans for CO2 storage differs for people living in the direct vicinity of a proposed CO2 storage location and people who do not.
Terwel B.W. [60]	2009	148	General	Public acceptance, trust	Questionnaire	to examine the influence of competence-based and integrity-based trust on public acceptance.
Toikka A. [133]	2014	25	General	Social and political issues of CCS	Interviews, workshops, media analysis, based of Eurobarometer	to map societal issues of CCS adoption based on multiple data sets from two research projects in Finland, looking at social, cultural, and political issues.
Tokushige K. [56]	2007	423	General	Public perception, acceptance	ICQ	to analyze and evaluate through a factor analysis how the general public perceives the CO2 geological storage, what factors are crucial to their acceptance of the storage, and what kinds of information would influence the acceptance and its factors.
Tokushige K. [58]	2006	267	General	Risk and benefit perception, acceptance	Questionnaire	to analyze and evaluate how the general public perceives CO2 geological storage technology among other global warming mitigation technologies and what factors are crucial for its acceptance; to analyze and evaluate what kind of information would influence public acceptance and notification.
Trinh H.A.N. [147]	2015	16	General	Experts' perception	Qualitative, Interviews	to summarize expert opinions regarding crucial factors that may influence Vietnam's future use of carbon capture and storage.
Upham P. [117]	2011	56	Cross-country	Public perception	Questionnaire	to determine European public perception of CCS.
Upham P. [65]	2011	N/A	Cross-country	Public perception	Focus groups, questionnaire	to describe the methods and results of six focus groups in different countries, aimed at developing communication strategies and media that are designed to briefly convey to stakeholders and the public the advantages and risks of CO2 capture and storage.
van Alphen K. [35]	2007	N/A	General	Public perception, mass media influence	Workshops, media analysis, interviews	to describe an extensive study on the acceptance of CCS by stakeholders in the Netherlands; to explore the influence of the way the Dutch press perceives and portrays CCS on the acceptance of CCS by the lay public.
van Os H.W.A. [131]	2013	55	General-Local	Public perception, role of stakeholders, NIMBY	Interviews	to explore the role of stakeholders responsibilities in the process of CCS project implementation.
Vercelli S. [15]	2013	N/A	General	Review	Qualitative, Literature review	to offer an overview of research bodies and provide useful criteria for its exploitation with regard to the interaction between information provision and public perception of CCS.
Vercelli S. [83]	2009	13 school classes, 13 researchers	General	Public perception, culture	Interviews, classes	to explore social representations and cultural models that could facilitate or hinder the necessary decisions for the implementation of Carbon Capture and Storage.
Vögele S. [150]	2018	0	General	Comparison of low carbon alternatives	Multi-criteria analysis, scenario assessment	to highlight reasons for CCS support descending in Germany and other European countries.
Wade S. [161]	2011	N/A	General	Social site characterization	Qualitative	to advance understanding of the subsurface and the technical, social and legal aspects of CCS.
Wallquist L. [138]	2010	654	General	Benefit and risk perception	Mail survey	to quantify laypeople's perception of critical aspects of CCS and to examine their impact on perceived benefits and perceived risks.
Wallquist L. [52]	2011	139	General	Preferences between CCS system elements, NIMBY	Online survey	to examine public preferences for the characteristics of the elements capture, transport, and storage in combination.
Wallquist L. [63]	2012	769	General	Trust, convictions, protest potential	Mail questionnaire	to examine the roles of trust and convictions for public attitude towards CCS to test a model that explains the public's protest potential against CCS.
Wallquist L. [23]	2011	297	General	Benefit and risk perception	Mail survey	to examine whether comprehensive information about CCS can have any influence on perceived risks and benefits.
Wallquist L. [61]	2009	16	General	Public perception	Interviews	to study how laypeople perceive CCS and which cognitions they hold with respect to this technique.
Wallquist L. [30]	2011	63	General	Public perception, knowledge	Questionnaire	to examine antecedents of risk and benefit perception of CCS by means.
Weber V. [158]	2018	N/A	General	Review	Qualitative	to review CCS Directive of the European Union.
Wong-Parodi G. [98]	2009	14	Local	Public perception	Focus group	to explore the views of communities that may be directly impacted by the siting of CCS.
Wong-Parodi G. [59]	2011	59	General-Local	Public perception	Interviews	to understand how to influence citizens' attitudes toward CCS in regions with significant dependence on energy sector.
Yang L. [64]	2016	349	General	Trust, public perception	Online survey, interviews	to explore the factors affecting public acceptance of CCS technologies in China.
Yu H. [80]	2018	2080	General	Preferences between technologies	Online questionnaire	to study public attitudes towards different low-carbon energy technologies, using nuclear power, CCS and wind energy as examples.

* Local level includes studies, based on areas with already started process of CCS project execution. General level includes surveys of all scale in one country, theoretical studies and reports.

** Questionnaire means paper-and-pencil questionnaire.

**Table:** 

Reference	Kn	Acc	BR	GovS	SD	Tr	Aw	NIMBY	Will
Anghel S. [46]									
Ashworth P. [18]									
Ashworth P. [31]									
Duetschke E. [53]									
Ter Mors E. [32]	+	+							
Fleishman L.A. [112]									
Hansson A. [145]									
Rychlicki S. [41]									
Schumann D. [151]									
Wallquist L. [23]	+	+	+						
Breukers S. [77]									
Brunsting S. [94]									
Buhr K. [44]									
Gough C. [95]				+	+	+			
Karayannis V. [92]									
Prangnell M. [140]									
Riesch H. [62]									
Yang L. [64]	+	+	+	+		+			
Ashworth P. [105]									
Mabon L. [86]									
Sharma S. [142]				+	+				
Chen Z.-A. [9]									
Li Q. [118]									
Wallquist L. [61]	+	+	+	+			+	+	
de Best-Waldhober M. [119]									
Itaoka K. [34]									
Jones C.R. [121]	+	+					+		
Gough C. [127]									
Karimi F. [3]									
Trinh H.A.N. [147]				+					
Lupion M. [143]									
Wallquist L. [63]									
Wong-Parodi G. [59]			+	+	+	+			
Ter Mors E. [129]									
Terwel B.W. [101]									
Terwel B.W. [103]				+		+			
Anderson C. [120]									
Oltra C. [153]			+	+	+	+		+	
Ashworth P. [99]									
Shackley S. [22]	+	+	+	+			+		
Boyd A.D. [139]									
Bradbury J. [76]	+		+		+	+	+		
Carley S.R. [47]									
Yu H. [80]	+	+	+		+	+			
Cherry T. L. [111]									
Mabon L. [26]		+	+	+	+				
de Best-Waldhober M. [113]									
Kraeusel J. [89]	+	+	+				+		+
de Best-Waldhober M. [33]									
Ha-Duong M. [148]	+	+			+		+		
de Coninck H. [141]									
Reiner D.M. [116]	+	+		+			+		
Desbarats J. [125]									
Terwel B.W. [97]	+	+	+	+	+	+	+	+	
Duetschke E. [155]									
Gough C. [36]	+			+		+			
Duetschke E. [37]									
Pietzner K. [39]	+	+			+				
Einsiedel E.F. [84]									
Tokushige K. [56]	+	+	+	+		+	+	+	
Mabon L. [146]									
Pihkola H. [144]		+		+					
Midden C.J.H. [49]									
Offermann-van Heek J. [122]			+			+			
Perdan S. [157]									
Wong-Parodi G. [98]	+		+		+			+	
Sacuta N. [106]									
Shackley S. [88]	+	+		+					
Arning K. [156]	+		+						
Ashworth P. [114]		+			+				
Billson M. [128]		+		+					+
Braun C. [149]	+	+			+	+			
Brunsting S. [160]	+	+	+	+	+	+			
Budinis S. [163]		+	+						
Chaudhry R. [126]	+	+	+	+					
de Bruin W.B. [45]	+								
Dowd A.-M. [38]	+	+		+	+	+	+		
Duan H. [87]	+	+		+	+		+		
Fischedick M. [4]			+	+				+	
Gough C. [132]	+		+	+		+	+		
Gough C. [67]		+		+			+	+	
Haug J.K. [135]	+	+	+	+	+				
Hope A.L.B. [82]		+	+		+		+		
Howell R. [24]		+	+	+	+	+			+
Itaoka K. [66]		+				+			
Itaoka K. [91]		+							+
Johnsson F. [104]		+	+	+					
Jouvet P.-A. [69]		+							
Kaiser M. [107]	+		+			+		+	
Karimi F. [68]	+		+		+	+	+	+	
Karimi F. [72]	+	+	+		+	+		+	
Karimi F. [73]	+		+		+		+		
Karimi F. [81]			+		+			+	
Klass A.B. [124]			+	+					
Krause R.M. [54]					+			+	
Kubota H. [19]	+		+	+			+		
Li Q. [28]	+	+	+				+	+	
Malone E.L. [42]	+	+		+	+	+			
Markusson N. [2]	+	+	+	+				+	
Miller E. [50]	+			+	+	+		+	
Miller E. [51]	+		+	+		+	+	+	
Moutenet J.-P. [43]	+		+	+	+		+		
Nicole M.A. [108]	+		+	+		+		+	
Oltra C. [16]		+	+	+		+			
Oltra C. [20]	+	+	+					+	
Palmgren C. [137]	+	+	+	+			+		+
Perdan S. [157]	+		+		+			+	
Reiner D. [115]	+	+		+					+
Sala R. [40]	+	+	+			+			
Seigo S.L. [29]	+		+	+					
Seigo S.L. [57]	+	+	+			+	+	+	
Seigo S.L. [75]	+		+		+				
Sharp J.D. [74]	+	+	+	+			+	+	+
Stephens J.C. [79]	+	+			+			+	
Ter Mors E. [21]	+			+	+				
Terwel B.W. [134]		+		+		+			
Terwel B.W. [55]		+	+	+	+	+		+	
Terwel B.W. [60]		+	+			+			
Toikka A. [133]		+		+	+				
Tokushige K. [58]		+	+					+	
Upham P. [117]	+	+	+	+		+	+	+	+
Upham P. [65]	+	+	+			+		+	
van Alphen K. [35]		+	+	+				+	
van Os H.W.A. [131]	+			+				+	
Vercelli S. [83]	+			+	+		+		
Vögele S. [150]			+	+		+		+	+
Wade S. [161]			+	+	+				
Wallquist L. [138]	+	+	+		+		+		
Wallquist L. [30]	+		+				+		
Wallquist L. [52]	+	+					+	+	

*Kn – Knowledge, Acc – Acceptance of CCS and preference between technologies, BR - Benefits and Risks perception, GovS - Governmental Policy and Interaction between Stakeholders, SD - Socio-demographic factors, Tr – Trust, Aw – Awareness, Will - Willingness to pay.

## References

[1] Tcvetkov P., Cherepovitsyn A. (2016). Prospects of CCS projects implementation in Russia: environmental protection and economic opportunities. J. Ecol. Eng..

[2] Markusson N., Shackley S. (2012). The Social Dynamic of Carbon Capture and Storage: Understanding CCS Representations, Governance and Innovation.

[3] Karimi F. (2017). Timescapes of CCS projects: is deferring projects and policies just kicking the can down the road?. Energy Procedia.

[4] Fischedick M., Pietzner K., Supersberger N., Esken A., Kuckshinrichs W., Zapp P., Gruber E. (2009). Stakeholder Acceptance of Carbon Capture and Storage in Germany.

[5] Selma L., Seigo O., Dohle S., Siegrist M. (2014). Public perception of carbon capture and storage (CCS): a review. Renew. Sustain. Energy Rev..

[6] Boyd E. (2009). Governing the Clean Development Mechanism: global rhetoric versus local realities in carbon sequestration projects. Environ. Plan..

[7] Visschers V.H.M., Keller C., Siegrist M. (2011). Climate change benefits and energy supply benefits as determinants of acceptance of nuclear power stations: investigating an explanatory model. Energy Policy.

[8] Visschers V.H.M., Siegrist M. (2012). Fair play in energy policy decisions: procedural fairness, outcome fairness and acceptance of the decision to rebuild nuclear power plants. Energy Policy.

[9] Chen Z.A., Li Q., Liu L.C., Zhang X., Kuang L., Jia L., Liu G. (2015). A large national survey of public perceptions of CCS technology in China. Appl. Energy.

[10] Cohen J.J., Reichl J., Schmidthaler M. (2014). Re-focussing research efforts on the public acceptance of energy infrastructure: a critical review. Energy.

[11] Pol E., Di Masso A., Castrechini A., Bonet M.R., Vidal T. (2006). Psychological parameters to understand and manage the NIMBY effect. Revue Européenne de Psychologie Appliquée/Eur. Rev. Appl. Psychol..

[12] Rousseau D.M., Sitkin S.B., Burt R.S., Camerer C. (1998). Not so different after all: a cross- discipline view of trust. Acad. Manag. Rev..

[13] Rowley J., Slack F. (2004). Conducting a literature review. Manag. Res. News.

[14] Statement by three national academies (Académie des Sciences, Leopoldina and Royal Society) on good practice in the evaluation of researchers and research programmes. URL: http://www.academie-sciences.fr/pdf/rapport/avis111217.pdf (accessed: 14.09.2018).

[15] Vercelli S., Anderlucci J., Memoli R., Battisti N., Mabon L., Lombardi S. (2013). Informing people about CCS: a review of social research studies. Energy Procedia.

[16] Oltra C., Sala R., Solà R., Di Masso M., Rowe G. (2010). Lay perceptions of carbon capture and storage technology. Int. J. Greenh. Gas Contr..

[17] Prades A., Horlick-Jones T., Oltra C., Sola` R. (2008). Lay perceptions of nuclear fusion: multiple modes of understanding. Sci. Public Policy.

[18] Ashworth P., Einsiedel E., Howell R., Brunsting S., Boughen N., Boyd A. (2013). Public preferences to CCS: how does it change across countries?. Energy Procedia.

[19] Kubota H., Shimota A. (2017). How should information about CCS be shared with the Japanese public?. Energy Procedia.

[20] Oltra C., Sala R., Boso À. (2012). The influence of information on individuals' reactions to CCS technologies: results from experimental online survey research. Greenh. Gases: Sci. Technol..

[21] ter Mors E., Weenig M.W.H., Ellemers N., Daamen D.D.L. (2010). Effective communication about complex environmental issues: perceived quality of information about carbon dioxide capture and storage (CCS) depends on stakeholder collaboration. J. Environ. Psychol..

[22] Shackley S., McLachlan C., Gough C. (2004). The Public Perceptions of Carbon Capture and Storage.

[23] Wallquist L., Visschers V.H., Dohle S., Siegrist M. (2011). Adapting communication to the public's intuitive understanding of CCS. Greenhouse Gases: Sci. Technol..

[24] Howell R., Shackley S., Mabon L., Ashworth P., Jeanneret T. (2014). Engaging the public with low-carbon energy technologies: results from a Scottish large group process. Energy Policy.

[25] Slovic P., Finucane M.L., Peters E., MacGregor D.G. (2007). The affect heuristic. Eur. J. Oper. Res..

[26] Mabon L., Shackley S., Bower-Bir N. (2014). Perceptions of sub-seabed carbon dioxide storage in Scotland and implications for policy: a qualitative study. Mar. Policy.

[27] Gigerenzer G. (2008). Why heuristics work. Perspect. Psychol. Sci..

[28] Li Q., Liu G., Leamon G., Liu L.C., Cai B., Chen Z.A. (2017). A national survey of public awareness of the environmental impact and management of CCUS technology in China. Energy Procedia.

[29] L'Orange Seigo S., Dohle S., Siegrist M. (2013). The effect of figures in CCS communication. Int. J. Greenh. Gas Contr..

[30] Wallquist L., Visschers V.H., Siegrist M. (2011). Antecedents of risk and benefit perception of CCS. Energy Procedia.

[31] Ashworth P., Carr-Cornish S., Boughen N., Thambimuthu K. (2009). Engaging the public on carbon dioxide capture and storage: does a large group process work?. Energy Procedia.

[32] ter Mors E., Terwel B.W., Daamen D.D., Reiner D.M., Schumann D., Anghel S. (2013). A comparison of techniques used to collect informed public opinions about CCS: opinion quality after focus group discussions versus information-choice questionnaires. Int. J. Greenh. Gas Contr..

[33] de Best-Waldhober M., Brunsting S., Paukovic M. (2012). Public concepts of CCS: understanding of the Dutch general public and its reflection in the media. Int. J. Greenh. Gas Contr..

[34] Itaoka K., Okuda Y., Saito A., Akai M. (2009). Influential information and factors for social acceptance of CCS: the 2nd round survey of public opinion in Japan. Energy Procedia.

[35] Van Alphen K., tot Voorst Q.V.V., Hekkert M.P., Smits R.E. (2007). Societal acceptance of carbon capture and storage technologies. Energy Policy.

[36] Gough C., Cunningham R., Mander S. (2017). Societal responses to CO2 storage in the UK: media, stakeholder and public perspectives. Energy Procedia.

[37] Dütschke E., Wohlfarth K., Höller S., Viebahn P., Schumann D., Pietzner K. (2016). Differences in the public perception of CCS in Germany depending on CO2 source, transport option and storage location. Int. J. Greenh. Gas Contr..

[38] Dowd A.M., Itaoka K., Ashworth P., Saito A., de Best-Waldhober M. (2014). Investigating the link between knowledge and perception of CO2 and CCS: an international study. Int. J. Greenh. Gas Contr..

[39] Pietzner K., Schumann D., Tvedt S.D., Næss R., Reiner D.M., Anghel S. (2011). Public Awareness and Perceptions of Carbon Dioxide Capture and Storage (CCS): Insights from Surveys Administered to Representative Samples in Six European Countries.

[40] Sala R., Oltra C. (2011). Experts’ attitudes towards CCS technologies in Spain. Int. J. Greenh. Gas Contr..

[41] Rychlicki S., Kosowski P., Wartak J., Solecki M. (2015). Social acceptance for CO2-EOR and CCS projects based on survey conducted in southeastern Poland. AGH Drilling, Oil, Gas.

[42] Malone E.L., Dooley J.J., Bradbury J.A. (2010). Moving from misinformation derived from public attitude surveys on carbon dioxide capture and storage towards realistic stakeholder involvement. Int. J. Greenh. Gas Contr..

[43] Moutenet J.-P., Bédard K., Malo M. (2012). Public awareness and opinion on CCS in the province of Québec, Canada. Greenh. Gases Sci. Technol..

[44] Buhr K., Wibeck V. (2014). Communication approaches for carbon capture and storage: underlying assumptions of limited versus extensive public engagement. Energy Res. Soc. Sci..

[45] Bruine de Bruin W., Wong-Parodi G. (2014). The role of initial affective impressions in responses to educational communications: the case of carbon capture and sequestration (CCS). J. Exp. Psychol. Appl..

[46] Anghel S. (2017). Impact of CCS communication on the general and local public in Romania-oltenia region. Energy Procedia.

[47] Carley S.R., Krause R.M., Warren D.C., Rupp J.A., Graham J.D. (2012). Early public impressions of terrestrial carbon capture and storage in a coal-intensive state. Environ. Sci. Technol..

[48] Itaoka K., Saito A. (2004). Akai M Public acceptance of CO2 capture and storage technology: a survey of public opinion to explore influential factors. Paper Presented for the 7th International Conference on Greenhouse Gas Control Technologies. Canada, Vancouver, September, 5–9.

[49] Midden C.J.H., Huijts N.M.A. (2009). The role of trust in the affective evaluation of novel risks: the case of CO 2 storage. Risk Anal..

[50] Miller E., Bell L., Buys L. (2007). Public understanding of carbon sequestration in Australia: socio-demographic predictors of knowledge, engagement and trust. Aust. J. Emerg. Technol. Soc..

[51] Miller E., Summerville J., Buys L., Bell L. (2008). Initial public perceptions of carbon geosequestration: implications for engagement and environmental risk communication strategies. Int. J. Glob Environ. Issues.

[52] Wallquist L., Seigo S.L.O., Visschers V.H., Siegrist M. (2012). Public acceptance of CCS system elements: a conjoint measurement. Int. J. Greenh. Gas Contr..

[53] Duetschke E., Schumann D., Pietzner K., Wohlfarth K., Höller S. (2014). Does it Make a Difference to the Public where CO2 Comes from and where it Is Stored?: an Experimental Approach to Enhance Understanding of CCS Perceptions.

[54] Krause R.M., Carley S.R., Warren D.C., Rupp J.A., Graham J.D. (2014). “Not in (or under) my backyard”: geographic proximity and public acceptance of carbon capture and storage facilities. Risk Anal..

[55] Terwel B.W., Daamen D.D.L. (2012). Initial public reactions to carbon capture and storage (CCS): differentiating general and local views. Clim. Policy.

[56] Tokushige K., Akimoto K., Tomoda T. (2007). Public perceptions on the acceptance of geological storage of carbon dioxide and information influencing the acceptance. Int. J. Greenh. Gas Contr..

[57] Seigo S.L.O., Arvai J., Dohle S., Siegrist M. (2014). Predictors of risk and benefit perception of carbon capture and storage (CCS) in regions with different stages of deployment. Int. J. Greenh. Gas Contr..

[58] Tokushige K., Akimoto K., Tomoda T. (2007). Public acceptance and risk-benefit perception of CO 2 geological storage for global warming mitigation in Japan. Mitig. Adapt. Strategies Glob. Change.

[59] Wong-Parodi G., Dowlatabadi H., McDaniels T., Isha X. (2011). Influencing attitudes toward carbon capture and sequestration: a social marketing approach. Environ. Sci. Technol..

[60] Terwel B.W., Harinck F., Ellemers N., Daamen D.D. (2009). Competence-based and integrity-based trust as predictors of acceptance of carbon dioxide capture and storage (CCS). Risk Anal.: Int. J..

[61] Wallquist L., Visschers V.H., Siegrist M. (2009). Lay concepts on CCS deployment in Switzerland based on qualitative interviews. Int. J. Greenh. Gas Contr..

[62] Riesch H., Oltra C., Lis A., Upham P., Pol M. (2013). Internet-based public debate of CCS: lessons from online focus groups in Poland and Spain. Energy Policy.

[63] Wallquist L., Visschers V.H., Dohle S., Siegrist M. (2012). The role of convictions and trust for public protest potential in the case of carbon dioxide capture and storage (CCS). Hum. Ecol. Risk Assess. Int. J..

[64] Yang L., Zhang X., McAlinden K.J. (2016). The effect of trust on people's acceptance of CCS (carbon capture and storage) technologies: evidence from a survey in the People's Republic of China. Energy.

[65] Upham P., Roberts T. (2011). Public perceptions of CCS in context: results of NearCO2 focus groups in the UK, Belgium, The Netherlands, Germany, Spain and Poland. Energy Procedia.

[66] Itaoka K., Saito A., Dowd A.M., de Best Waldhober M., Ashworth P. (2014). Influence of the large earthquake and nuclear plant accident on perception of CCS. Energy Procedia.

[67] Gough C., Taylor I., Shackley S. (2002). Burying carbon under the sea: an initial exploration of public opinions. Energy Environ..

[68] Karimi F., Toikka A. (2018). General public reactions to carbon capture and storage: does culture matter?. Int. J. Greenh. Gas Contr..

[69] Jouvet P.A., Renner M. (2015). Social acceptance and optimal pollution: CCS or tax?. Environ. Model. Assess..

[70] Schumann D., Kuckshinrichs W., Hake J.-F. (2014). Public acceptance. Carbon Capture, Storage and Use – Technical, Economic, Environmental and Societal Perspectives.

[71] Ashworth P., Wade S., Reiner D., Liang X. (2015). Developments in public communications on CCS. Int. J. Greenh. Gas Contr..

[72] Karimi F., Komendantova N. (2017). Understanding experts’ views and risk perceptions on carbon capture and storage in three European countries. Geojournal.

[73] Karimi F., Toikka A. (2014). The relation between cultural structures and risk perception: how does social acceptance of carbon capture and storage emerge?. Energy Procedia.

[74] Sharp J.D., Jaccard M.K., Keith D.W. (2009). Anticipating public attitudes toward underground CO 2 storage. Int. J. Greenh. Gas Contr..

[75] L'Orange Seigo S., Wallquist L., Dohle S., Siegrist M. (2011). Communication of CCS monitoring activities may not have a reassuring effect on the public. Int. J. Greenh. Gas Contr..

[76] Bradbury J., Ray I., Peterson T., Wade S., Wong-Parodi G., Feldpausch A. (2009). The role of social factors in shaping public perceptions of CCS: results of multi-state focus group interviews in the US. Energy Procedia.

[77] Breukers S., Upham P. (2015). Organisational aspects of public engagement in European energy infrastructure planning: the case of early-stage CCS projects. J. Environ. Plan. Manag..

[78] Farhar B.C., Unseld C.T., Vories R., Crews R. (1980). Public opinion about energy. Annu. Rev. Energy.

[79] Stephens J.C., Bielicki J., Rand G.M. (2009). Learning about carbon capture and storage: changing stakeholder perceptions with expert information. Energy Procedia.

[80] Yu H., Reiner D., Chen H., Mi Z. (2018). A Comparison of Public Preferences for Different Low-Carbon Energy Technologies: Support for CCS, Nuclear and Wind Energy in the United Kingdom.

[81] Karimi F., Toikka A., Hukkinen J.I. (2016). Comparative socio-cultural analysis of risk perception of carbon capture and storage in the European union. Energy Res. Soc. Sci..

[82] Hope A.L., Jones C.R. (2014). The impact of religious faith on attitudes to environmental issues and Carbon Capture and Storage (CCS) technologies: a mixed methods study. Technol. Soc..

[83] Vercelli S., Lombardi S. (2009). CCS as part of a global cultural development for environmentally sustainable energy production. Energy Procedia.

[84] Einsiedel E.F., Boyd A.D., Medlock J., Ashworth P. (2013). Assessing socio-technical mindsets: public deliberations on carbon capture and storage in the context of energy sources and climate change. Energy Policy.

[85] Barnett J., Burningham K., Walker G., Cass N. (2012). Imagined publics and engagement around renewable energy technologies in the UK. Public Underst. Sci..

[86] Mabon L., Vercelli S., Shackley S., Anderlucci J., Battisti N., Franzese C., Boot K. (2013). ‘Tell me what you think about the geological storage of carbon dioxide’: towards a fuller understanding of public perceptions of CCS. Energy Procedia.

[87] Duan H. (2010). The public perspective of carbon capture and storage for CO 2 emission reductions in China. Energy Policy.

[88] Shackley S., Reiner D., Upham P., de Coninck H., Sigurthorsson G., Anderson J. (2009). The acceptability of CO2 capture and storage (CCS) in Europe: an assessment of the key determining factors: Part 2. The social acceptability of CCS and the wider impacts and repercussions of its implementation. Int. J. Greenh. Gas Contr..

[89] Kraeusel J., Möst D. (2012). Carbon Capture and Storage on its way to large-scale deployment: social acceptance and willingness to pay in Germany. Energy Policy.

[90] Eurobarometer S. (2009). Europeans’ attitudes towards climate change. Eur. Comm..

[91] Itaoka K., Saito A., Akai M. (2017). Policy parity for CCS?–Public preference on low carbon electricity. Energy Procedia.

[92] Karayannis V., Charalampides G., Lakioti E. (2014). Socio-economic aspects of CCS technologies. Procedia Economics and Finance.

[93] Hanemann W.M. (1991). Willingness to pay and willingness to accept: how much can they differ?. Am. Econ. Rev..

[94] Brunsting S., Desbarats J., de Best-Waldhober M., Duetschke E., Oltra C., Upham P., Riesch H. (2011). The public and CCS: the importance of communication and participation in the context of local realities. Energy Procedia.

[95] Gough C., Cunningham R., Mander S. (2018). Understanding key elements in establishing a social license for CCS: an empirical approach. Int. J. Greenh. Gas Contr..

[96] Huijts N.M.A., Molin E.J.E., Steg L. (2012). Psychological factors influencing sustainable energy technology acceptance: a review-based comprehensive framework. Renew. Sustain. Energy Rev..

[97] Terwel B.W., ter Mors E., Daamen D.D.L. (2012). It's not only about safety: beliefs and attitudes of 811 local residents regarding a ccs project in Barendrecht. Int. J. Greenh. Gas Contr..

[98] Wong-Parodi G., Ray I. (2009). Community perceptions of carbon sequestration: insights from California. Environ. Res. Lett..

[99] Ashworth P., Boughen N., Mayhew M., Millar F. (2009). An integrated roadmap of communication activities around carbon capture and storage in Australia and beyond. Energy Procedia.

[100] Bäckstrand K., Meadowcroft J., Oppenheimer M. (2011). The Politics and Policy of Carbon Capture and Storage: Framing an Emergent Technology.

[101] Terwel B.W., Harinck F., Ellemers N., Daamen D.D. (2011). Going beyond the properties of CO2 capture and storage (CCS) technology: how trust in stakeholders affects public acceptance of CCS. Int. J. Greenh. Gas Contr..

[102] De Vries G., Terwel B.W., Ellemers N., Daamen D.D. (2015). Sustainability or profitability? How communicated motives for environmental policy affect public perceptions of corporate greenwashing. Corp. Soc. Responsib. Environ. Manag..

[103] Terwel B.W., Harinck F., Ellemers N., Daamen D.D.L. (2009). How organizational motives and communications affect public trust in organizations: the case of carbon dioxide capture and storage. J. Environ. Psychol..

[104] Johnsson F., Reiner D., Itaoka K., Herzog H. (2010). Stakeholder attitudes on carbon capture and storage—an international comparison. Int. J. Greenh. Gas Contr..

[105] Ashworth P., Bradbury J., Wade S., Feenstra C.F.J.Y., Greenberg S., Hund G. (2012). What's in store: lessons from implementing CCS. Int. J. Greenh. Gas Contr..

[106] Sacuta N., Daly D., Botnen B., Worth K. (2017). Communicating about the geological storage of carbon dioxide–comparing public outreach for CO2 EOR and saline storage projects. Energy Procedia.

[107] Kaiser M., Zimmer R., Brunsting S., Mastop J., Pol M. (2014). Development of CCS projects in Poland. How to communicate with the local public?. Energy Procedia.

[108] Huijts N.M., Midden C.J., Meijnders A.L. (2007). Social acceptance of carbon dioxide storage. Energy Policy.

[109] Huijts N.M.A., Molin E.J.E., Steg L. (2012). Psychological factors influencing sustainable energy technology acceptance: a review-based comprehensive framework. Renew. Sustain. Energy Rev..

[110] Batel S., Devine-Wright P., Tangeland T. (2013). Social acceptance of low carbon energy and associated infrastructures: a critical discussion. Energy Policy.

[111] Cherry T.L., García J.H., Kallbekken S., Torvanger A. (2014). The development and deployment of low-carbon energy technologies: the role of economic interests and cultural worldviews on public support. Energy Policy.

[112] Fleishman L.A., De Bruin W.B., Morgan M.G. (2010). Informed public preferences for electricity portfolios with CCS and other low-carbon technologies. Risk Anal.: Int. J..

[113] de Best-Waldhober M., Daamen D., Faaij A. (2009). Informed and uninformed public opinions on CO 2 capture and storage technologies in The Netherlands. Int. J. Greenh. Gas Contr..

[114] Ashworth P., Pisarski A., Thambimuthu K. (2009). Public acceptance of carbon dioxide capture and storage in a proposed demonstration area. Proc. Inst. Mech. Eng. Part A J Power Energy.

[115] Reiner D., Curry T., de Figueiredo M., Herzog H., Ansolabehere S., Itaoka K. (2006). An International Comparison of Public Attitudes towards Carbon Capture and Storage Technologies. http://www.ghgt8.no.

[116] Reiner D.M., Curry T.E., De Figueiredo M.A., Herzog H.J., Ansolabehere S.D., Itaoka K. (2006). American exceptionalism? Similarities and differences in national attitudes toward energy policy and global warming. Environ. Sci. Technol..

[117] Upham P., Roberts T. (2011). Public perceptions of CCS: emergent themes in pan-European focus groups and implications for communications. Int. J. Greenh. Gas Contr..

[118] Li Q., Liu L.C., Chen Z.A., Zhang X., Jia L., Liu G. (2014). A survey of public perception of CCUS in China. Energy Procedia.

[119] de Best-Waldhober M., Daamen D., Ramirez A.R., Faaij A., Hendriks C., de Visser E. (2012). Informed public opinion in The Netherlands: evaluation of CO 2 capture and storage technologies in comparison with other CO 2 mitigation options. Int. J. Greenh. Gas Contr..

[120] Anderson C., Schirmer J., Abjorensen N. (2012). Exploring CCS community acceptance and public participation from a human and social capital perspective. Mitig. Adapt. Strategies Glob. Change.

[121] Jones C.R., Radford R.L., Armstrong K., Styring P. (2014). What a waste! Assessing public perceptions of Carbon Dioxide Utilisation technology. J. CO2 Util..

[122] van Heek J., Arning K., Linzenich A., Ziefle M. (2018). Trust and distrust in Carbon Capture and Utilization industry as relevant factors for the acceptance of carbon-based products. Front. Energy Res..

[123] Sjöberg L. (2000). Perceived risk and tampering with nature. J. Risk Res..

[124] Klass A.B., Wilson E.J. (2008). Climate change and carbon sequestration: assessing a liability regime for long-term storage of carbon dioxide. Emory LJ..

[125] Desbarats J., Upham P., Riesch H., Reiner D., Brunsting S., de Best-Waldhober M. (2010). Review of the public participation practices for CCS and non-CCS projects in Europe.

[126] Chaudhry R., Fischlein M., Larson J., Hall D.M., Peterson T.R., Wilson E.J., Stephens J.C. (2013). Policy stakeholders' perceptions of carbon capture and storage: a comparison of four US States. J. Clean. Prod..

[127] Gough C., Mander S., Haszeldine S. (2010). A roadmap for carbon capture and storage in the UK. Int. J. Greenh. Gas Contr..

[128] Billson M., Pourkashanian M. (2017). The evolution of European CCS policy. Energy Procedia.

[129] ter Mors E., Weenig M.W., Ellemers N., Daamen D.D., de Best-Waldhober M. (2009). Public information: on why and when multiple information sources are more effective than single information sources in communication about CCS. Energy Procedia.

[130] Gross C. (2007). Community perspectives of wind energy in Australia: the application of a justice and community fairness framework to increase social acceptance. Energy Policy.

[131] van Os H.W., Herber R., Scholtens B. (2014). Not Under Our Back Yards? A case study of social acceptance of the Northern Netherlands CCS initiative. Renew. Sustain. Energy Rev..

[132] Gough C., Mander S. (2014). Public perceptions of CO2 transportation in pipelines. Energy Policy.

[133] Toikka A.I., Kojo M., Kainiemi L. (2014). What is the socio-political scaffolding CCS needs to thrive?. Energy Procedia.

[134] Terwel B.W., Harinck F., Ellemers N., Daamen D.D.L. (2010). Voice in political decision making: the effect of group voice on perceived trustworthiness of decision makers and subsequent acceptance of decisions. J. Exp. Psychol. Appl..

[135] Haug J.K., Stigson P. (2016). Local acceptance and communication as crucial elements for realizing CCS in the Nordic region. Energy Procedia.

[136] Cherepovitsyn A., Tcvetkov P. (2017). Overview of the prospects for developing a renewable energy in Russia. Proceedings of 2017 International Conference on Green Energy and Applications, ICGEA.

[137] Palmgren C.R., Morgan G.M., Bruine de Bruin W., Keith D.W. (2004). Initial public perceptions of deep geological and oceanic disposal of carbon dioxide. Environ. Sci. Technol..

[138] Wallquist L., Visschers V.H.M., Siegrist M. (2010). Impact of knowledge and misconceptions on benefit and risk perception of ccs. Environ. Sci. Technol..

[139] Boyd A.D., Hmielowski J.D., David P. (2017). Public perceptions of carbon capture and storage in Canada: results of a national survey. Int. J. Greenh. Gas Contr..

[140] Prangnell M. (2013). Communications for Carbon Capture and Storage: Identifying the Benefits, Managing Risk and Maintaining the Trust of Stakeholders.

[141] de Coninck H., Stephens J.C., Metz B. (2009). Global learning on carbon capture and storage: a call for strong international cooperation on CCS demonstration. Energy Policy.

[142] Sharma S., Cook P., Robinson S., Anderson C. (2007). Regulatory challenges and managing public perception in planning a geological storage pilot project in Australia. Int. J. Greenh. Gas Contr..

[143] Lupion M., Pérez A., Torrecilla F., Merino B. (2013). Lessons learned from the public perception and engagement strategy-experiences in CIUDEN's CCS facilities in Spain. Energy Procedia.

[144] Pihkola H., Tsupari E., Kojo M., Kujanpää L., Nissilä M., Sokka L., Behm K. (2017). Integrated sustainability assessment of CCS–identifying non-technical barriers and drivers for CCS implementation in Finland. Energy Procedia.

[145] Hansson A., Bryngelsson M. (2005). Attitudes Regarding CO2 Capture and Storage from a Swedish Perspective.

[146] Mabon L., Littlecott C. (2015). CO₂-EOR Stakeholder Perceptions and Policy Responses.

[147] Nguyen-Trinh H.A., Ha-Duong M. (2015). Perspective of CO2 capture & storage (CCS) development in Vietnam: results from expert interviews. Int. J. Greenh. Gas Contr..

[148] Ha-Duong M., Nadaï A., Campos A.S. (2009). A survey on the public perception of CCS in France. Int. J. Greenh. Gas Contr..

[149] Braun C., Merk C., Pönitzsch G., Rehdanz K., Schmidt U. (2018). Public perception of climate engineering and carbon capture and storage in Germany: survey evidence. Clim. Policy.

[150] Vögele S., Rübbelke D., Mayer P., Kuckshinrichs W. (2018). Germany’s “No” to carbon capture and storage: just a question of lacking acceptance?. Appl. Energy.

[151] Schumann D., Duetschke E., Pietzner K. (2014). Public Perception of CO2 Offshore Storage in Germany: Regional Differences and Determinants.

[152] Schumann D. (2017). Public perception of CO2 pipelines. Energy Procedia.

[153] Oltra C., Upham P., Riesch H., Boso A., Brunsting S., Dütschke E. (2012). Public responses to CO 2 storage sites: lessons from five European cases. Energy Environ..

[154] Lockwood T. (2017). Public Outreach Approaches for Carbon Capture and Storage Projects.

[155] Dütschke E. (2011). What drives local public acceptance–comparing two cases from Germany. Energy Procedia.

[156] Arning K., van Heek J., Ziefle M. (2017). Risk perception and acceptance of CDU consumer products in Germany. Energy Procedia.

[157] Perdan S., Jones C.R., Azapagic A. (2017). Public awareness and acceptance of carbon capture and utilisation in the UK. Sustain. Prod. Consum..

[158] Weber V. (2018). Uncertain liability and stagnating CCS deployment in the European union: is it the member states’ turn? Review of European. Comp. Int. Environ. Law.

[159] Wüstenhagen R., Wolsink M., Bürer M.J. (2007). Social acceptance of renewable energy innovation: an introduction to the concept. Energy Policy.

[160] Brunsting S., Pol M., Mastop J., Kaiser M., Zimmer R., Shackley S. (2013). Social Site Characterisation for CO2 storage operations to inform public engagement in Poland and Scotland. Energy Procedia.

[161] Wade S., Greenberg S. (2011). Social site characterisation: from concept to application. A Review of Relevant Social Science Literature and a Toolkit for Social Site Characterisation.

[162] Tcvetkov P., Cherepovitsyn A., Fedoseev S. (2019). The changing role of CO2 in the transition to a circular economy: review of carbon sequestration projects. Sustainability.

[163] Cherepovitsyn A., Fedoseev S., Tcvetkov P., Sidorova K., Kraslawski A. (2018). Potential of Russian regions to implement CO2-enhanced oil recovery. Energies.

[164] Budinis S., Krevor S., Dowell N.M., Brandon N., Hawkes A. (2018). An assessment of CCS costs, barriers and potential. Energy Strategy Rev..

[165] Kozlov A., Teslya A., Chernogorskii S. (2019). Game theory model of state investment into territories of advanced development in the regions of mineral resources specialization. J. Min. Inst..

[166] Van Voorhees R.F. (2017). Crediting carbon dioxide storage associated with enhanced oil recovery. Energy Procedia.

